# The impact of family-related psychosocial factors on suicidal ideation among adolescents: a systematic review

**DOI:** 10.1186/s12889-026-27928-7

**Published:** 2026-07-09

**Authors:** Susmita Biswas, Urvi Shukla, Sucharita Maji, Gerald Jordan, Karl Andriessen

**Affiliations:** 1 Indian Institute of Technology (Indian School of Mines) Dhanbad, Dhanbad, India; 2 University of Birmingham, Birmingham, England; 3 School of Psychological Sciences, University of Bristol, Bristol, England; 4 The University of Melbourne, Melbourne, Australia

**Keywords:** Suicidal ideation, Family, Psychosocial factors, Adolescents, Risk factors, Protective factors

## Abstract

**Background:**

This systematic literature review investigates the impact of family-related psychosocial factors on suicidal ideation. Adolescents experiencing family-related adversities are more likely to experience suicidal ideation. Given that adolescents are a high-risk population, understanding the nature of the impact of family-related factors is important. It would help determine and differentiate the family-specific risk and protective factors and aid in designing targeted suicide prevention strategies.

**Method:**

The review followed Preferred Reporting Items for Systematic Reviews and Meta-Analyses guidelines. Systematic searches of peer-reviewed quantitative studies were conducted in Medline, PsycINFO, CINAHL, Scopus, and Web of Science databases. Due to the heterogeneity of the findings, the data were analyzed using narrative synthesis. (PROSPERO registration: CRD420250625290).

**Results:**

The review identified 94 studies published between 1993 and March 2025. The findings indicate that adolescents who experienced hostility inside the family, lacked healthy communication with family members, had controlling parents, and dealt with mental health issues of their parents had a high risk of suicidal ideation. Those who were satisfied with their family members, experienced parental emotional support, and had authoritative parents had a low risk of suicidal ideation.

**Discussion:**

The findings of the review provide insight into the diverse domains of family-related psychosocial factors holistically and highlight their dynamic nature. It suggests that some strategies, such as early diagnosis of parental mental health issues, encouraging open communication between parents and adolescents, and implementing work-life balance for parents, could help reduce suicidal ideation. The review was limited to quantitative studies; hence, further exploration of qualitative, longitudinal, and intervention-based studies may provide further understanding of familial influences on suicidal ideation in adolescents.

**Supplementary Information:**

The online version contains supplementary material available at https://doi.org/10.1186/s12889-026-27928-7.

## Introduction

Suicide is defined as the intentional demise caused by self-inflicted injurious behavior [1]. It is a global public health concern, with about an estimated 727,000 deaths by suicide every year [2]. It is also ranked to be the fourth leading cause of death for 15–19-year olds in both sexes globally [2]. Suicidal behavior is understood as a process that may include suicidal ideation and/or suicide attempts. The process may involve planning as well as impulsive acts [3].

Suicidal ideation is the desire or thought of ending one's own life [4]. The intensity of suicidal ideation exists either as a passive death wish without any active plan, or a death wish accompanied by an active plan [4, 5]. However, it may also transition from passive thoughts to active thoughts. Theoretical frameworks on suicidal behavior explain the transition from suicidal ideation to behavior as a complex interaction between several factors, such as psychological, biological, environmental, societal, and cultural.

Several psychological models of suicidal behavior have incorporated suicidal ideation as a core element within the broader suicidal process. In the stress-diathesis model, for instance, suicidal ideation is viewed as emerging when an individual with pre-existing vulnerabilities (genetic, biological, and psychological) faces extreme stress and is unable to cope with it [6]. Within the ideation-to-action framework, suicidal ideation is distinguished from behavior. It emphasizes on additional factors (increased pain tolerance and reduced fear of death) that determine the transition from ideation to action [7, 8]. Furthermore, the integrated motivational-volitional model (IMV) outlines three phases and positions suicidal ideation in the motivational phase, arising from defeat and feelings of entrapment [9]. These models collectively highlight suicidal ideation as a central stage in the suicidal process. These frameworks, although they identify suicidal ideation as a result of the interaction between individual vulnerabilities and immediate stressors, fail to specify the stressors or factors responsible at a concrete level [10]. Additionally, these models are predominantly derived from cross-sectional data, examining a sample of healthy young adults, and thus underexplores developmental variations in different high-risk populations, such as adolescents [11]. Despite the limitations, these frameworks highlight and emphasize the need for early identification of suicidal ideation and designing appropriate interventions, further helping to reduce suicidal risk [12].

Although suicide is a global concern among all age groups, understanding this behavior among adolescents requires immediate attention [11, 13]. The adolescent period is marked by significant changes, including physical and mental development [14], during which experiences related to life, relationships, and individuality are explored [15]. These changes are associated with growth but may also increase the risk of mental health problems and suicidal behavior [13]. Additionally, the risk of suicidal behavior may increase dramatically during the adolescent period, and about one-third of those who develop suicidal ideation further develop an active suicidal plan [16]. The factors that contribute to suicidal vulnerabilities are embedded in psychosocial components, including psychological, social, and family-related stressors. These factors may impact suicidal ideation and behavior either directly, indirectly, or in a combined pattern. Therefore, identifying such factors may help in reducing the progression of suicidal risk at an early stage [13].

Psychosocial factors may be defined as the interaction between an individual’s social environment and psychological processes that impact their emotional and mental health [17]. These factors include protective psychosocial resources (e.g., social support and social network) and psychological risk factors (e.g., hopelessness, depression, hostility, mental exhaustion) [17]. Within the domain of psychosocial factors, family-related factors hold significant value for adolescent growth and mental well-being [13]. A family setting provides a source where daily interactions, expectations, and norms are formed, and beliefs about safety, belonging, and mattering are fostered. Family-related psychosocial factors encompass emotional climate, family functioning, and patterns among family members [13, 18]. Studies on family factors and adolescent suicidal behavior suggest that supportive parental behavior during the relatively unstable period of adolescence is protective against developing suicidal ideation [18, 19]. Nonetheless, adolescents already experiencing adversities or setbacks during this period, and a lack of emotional support from their families, are likely to develop suicidal ideation or attempt suicide [13]. Therefore, family and parental support appear to be crucial for protecting adolescents from suicidal ideation [20].

The existing literature clearly emphasizes the importance of family-related psychosocial factors in understanding adolescent suicidal ideation; however, a systematic literature review entailing the impacts of family-related psychosocial factors on adolescent suicidal ideation is lacking. Existing reviews have explored broad psychosocial factors such as depression, anxiety, peer conflicts, bullying, and social support for suicidal ideation or suicide attempts, or both [21–24]. Other reviews have focused on different areas, such as social anxiety [25], or explain the prevalence of suicidal ideation [26]. None of these reviews have examined family-related factors associated with suicidal ideation in adolescents specifically; thus, this underlines a significant gap in understanding the impact of different types of family-related psychosocial factors on adolescent suicidal ideation. Addressing this gap will help youth, family members, clinicians, and policy makers better understand the importance of family in suicide, leading to better treatment across contexts. This is particularly relevant in Western contexts, where the role of family in care for serious mental health conditions more broadly is minimized, with potential negative effects [27].

Therefore, this review aims to systematically synthesize quantitative studies examining family-related psychosocial factors (family conflict, family functioning, parental mental health, parenting styles, parental control) that are associated with adolescent suicidal ideation as the primary outcome. The quantitative-only focus enables standardized outcome measurement and narrative synthesis using a vote-counting procedure, ensuring methodological consistency [28]. This review also focused on clarifying effect sizes, identifying moderators, and mediators to design accurate intervention targets for suicide prevention.

## Method

The review followed Preferred Reporting Items for Systematic Reviews and Meta-Analysis guidelines [29], and the research questions were formulated as per the PICO framework [30]. The framework was used to identify the Population (adolescents aged 10–19 years), Intervention (family-related psychosocial factors), Comparison (comparison among influencing factors), and Outcomes (adolescent suicidal ideation). This further guided the design of the search strategy and the development of inclusion/exclusion criteria. The review protocol was a priori registered in PROSPERO (CRD420250625290).

### Inclusion and exclusion criteria

Studies were included if: (i) the population consisted of adolescents (aged 10–19 years); (ii) they provided data on suicidal ideation associated with family-related psychosocial factors; (iii) used only quantitative methods; and (iv) were a primary, empirical study published in English in a peer-reviewed journal.

Quantitative studies were included exclusively to establish methodological consistency across studies. The review focused on synthesizing empirically measurable outcomes and their statistical associations.

Studies were excluded when: (i) more than half of the study sample was younger than age 10 or older than age 19, or the mean age of the sample was outside the 10–19 age range; (ii) the study did not provide data on suicidal ideation; (iii) the paper was not an empirical primary study, such as a systematic review or case report; and (iv) the article was not available in full text, English language or published in peer-review journal.

Mixed-method and qualitative studies were excluded due to their methodological heterogeneity, which could limit consistency in data extraction and synthesis. Further, mixed-method studies are usually designed to triangulate findings from quantitative and qualitative data. Including only quantitative data in the review would introduce bias, as the study's findings are based on a combination of quantitative and qualitative data. Additionally, mixed-methods and qualitative study analyses were beyond the scope of this review. This exclusion may have led to a lack of contextual understanding provided by qualitative studies.

### Search strategy

Two authors, SB and US, conducted searches on March 21, 2025, across five databases: Medline, PsycINFO, CINAHL, Scopus, and Web of Science. The search strings comprised three concepts: adolescents, family-related psychosocial factors, and suicidal ideation. The Medline and PsycINFO databases were searched, with a combination of MeSH terms and keywords to design the search string. The Medline search string included: (Adolescent/OR Students/OR Minors/OR adolescen*.mp. OR teen*.mp. OR youth*.mp. OR pupil*.mp. OR school-age* youth.mp. OR minor*.mp. OR underage*.mp. OR student*.mp.) AND (Suicidal Ideation/OR suicid* Ideation.mp. OR suicid* thought*.mp. OR suicidality.mp.) AND (Psychology/OR Family Relations/OR Parent–Child Relations/OR Child Abuse/OR Family Conflict/OR Parenting/OR Family/OR psychosoci* factor*.mp. OR famil* factor*.mp. OR famil*context*.mp. OR famil*dynamic*.mp. OR abus*.mp. OR famil* abus*.mp. ORparent* abus*.mp. OR famil* conflict*.mp. OR parent* support.mp. OR parent* style*.mp. OR family stress.mp. OR family dysfunction*.mp. OR neglect*.mp. OR parent*neglect*.mp. OR parent*.mp.). Similar search strings were applied for the other databases. The searches were limited to articles published in English-language and peer-reviewed journals, and not by publication date or location.

### Screening and data extraction

**Fig. 1:**
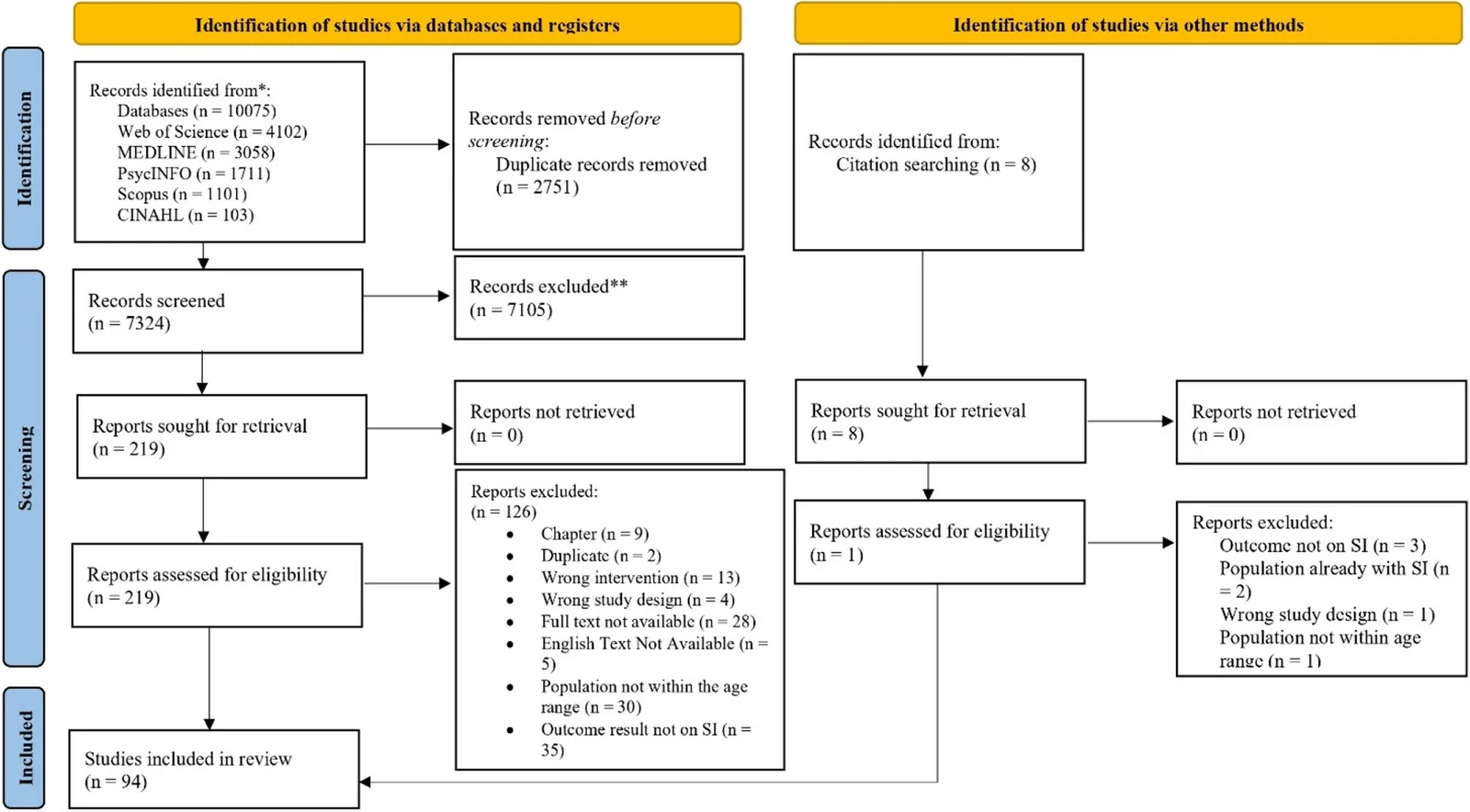
PRISMA flow diagram

Researcher SB imported the studies into the Covidence platform, a systematic review tool [31], and the records were deduplicated. Initially, SB and US independently screened 10% of the total studies based on their titles and abstracts to assess their potential eligibility [32]. Cohen’s kappa was used to assess inter-rater reliability (kappa = 0.63), indicating substantial agreement between the researchers. Then, SB screened the rest of the articles based on their titles and abstracts, following which SB and US independently screened the full texts of the selected articles based on the inclusion and exclusion criteria. The inter-rater reliability was almost perfect (kappa value = 0.98), with only two disagreements, which SM resolved when consulted. Figure 1 details the search and selection process.

The research team developed a data extraction form, including the following variables: author, sample size, gender, age, study design, family-related psychosocial factors, measures, and main findings detailing the risk and protective family-related psychosocial factors for adolescent SI, as specified in the study. Researchers SB and US independently extracted the data. Any discrepancies were resolved through discussion.

### Quality assessment

The quality and risk of bias of the included studies were assessed using the Joanna Briggs Institute (JBI) critical appraisal checklists [33] for cross-sectional studies, cohort studies, and randomized controlled trials. The checklist evaluated the quality of the studies based on their inclusion criteria, validity, and reliability of exposure and outcome factors, as well as the identification and management of confounding variables and the appropriateness of statistical analysis. Two researchers, SB and US, independently assessed the quality of the studies and rated them ‘yes’, ‘no’, ‘unclear’, or ‘not applicable’. The quality was judged based on the overall pattern of ratings, rather than a summary score aligning with JBI guidelines. The checklist allows the studies to be assessed based on individual appraisal items, rather than resorting to a scoring system (see Supplementary Material 2). The disagreements were resolved through mutual discussion, and a senior author was consulted. No study was excluded based on appraisal ratings because we wanted to have a full picture of the evidence in this review, including the overall quality of the evidence, which will support better methodological practices in the future.

### Data synthesis

A narrative synthesis approach was employed due to the heterogeneous nature of the included studies, which limited the use of statistical pooling in meta-analysis. We followed the Synthesis Without Meta-analysis (SWiM) guidelines to synthesize the quantitative data [34]. Studies were grouped based on similar exposure factors, and the outcomes were then explained in terms of the direction of effect or odds ratio, risk ratio, or reported significance, as applicable. Rather than a pooled summary effect, differences between studies and their statistical analysis were considered while interpreting patterns in the findings across studies.

## Results

### Study characteristics

The searches yielded 94 quantitative studies published from 1993 to 2025. The studies published in the years mentioned (Table 1 in Supplementary Material 3) and the geographical regions varied (Table 2 in Supplementary Material 3). The highest number of studies was published in 2023 (*n* = 13), and none were published in 2000, 2002, 2003, or 2007. The extracted data from the studies are available in Supplementary Material 1.

**Table 1:** Year (number of studies), included studies

Year (n)	Included Studies
2025 (2)	[35, 36]
2024 (8)	[37–44]
2023 (13)	[45–57]
2022 (9)	[58–66]
2021 (8)	[67–74]
2020 (9)	[75–83]
2019 (2)	[84, 85]
2018 (4)	[86–89]
2017 (4)	[90–93]
2016 (5)	[94–98]
2015 (3)	[99–101]
2014 (3)	[102–104]
2013 (2)	[105, 106]
2012 (3)	[107–109]
2011 (2)	[110, 111]
2010 (4)	[112–115]
2009 (4)	[116–119]
2008 (2)	[120, 121]
2007 (0)	
2006 (1)	[122]
2005 (3)	[123–125]
2004 (1)	[126]
2003 (0)	
2002 (0)	
2001 (1)	[127]
2000 (0)	
1993 (1)	[128]

**Table 2:** Region, country, and included studies

Region	Country	Included Studies
North America (19)	USA (18)	[36, 37, 49, 70, 73, 81, 84, 88, 89, 94, 101, 102, 111, 115, 119, 123, 124, 128]
	Canada (1)	[114]
	Saint Lucia (1)	[54]
South America (2)	Chile (1)	[80]
	Colombia (1)	[65]
Europe (12)	10 European countries (1)	[90]
	France (1)	[77]
	Spain (6)	[39, 43, 46, 50, 52, 63]
	Netherlands (1)	[121]
	Estonia (1)	[116]
	Portugal (2)	[97, 103]
Asia (53)	South Korea (10)	[56, 67, 73, 75, 76, 82, 86, 100, 113, 125]
	China (28)	[35, 40–42, 44, 45, 47, 48, 51, 57–62, 64, 66, 69, 78, 79, 91, 95, 99, 105, 110, 117, 118, 122]
	Hong Kong (3)	[74, 120, 127]
	Taiwan (1)	[104]
	Vietnam (1)	[98]
	Malaysia (3)	[83, 85, 96]
	Singapore (1)	[126]
	Israel (2)	[87, 109]
	Thailand (1)	[107]
	Saudi Arabia (1)	[38]
	Japan (1)	[72]
	Lebanon (1)	[112]
Africa (6)	South Africa (2)	[106, 108]
	Ethiopia (1)	[55]
	Morocco (1)	[92]
	Sierra Leone (1)	[68]
	Namibia (1)	[53]
Oceania (1)	Samoa (1)	[93]

### Sample

Across the 94 studies, the sample sizes ranged from *n* = 111 [70] to *n* = 72,435 [100]. Most studies involved almost an equal number of males and females, except for two, which solely focused on females [84, 102]. Additionally, two studies [36, 43] reported the gender identity of the participants over the sex assigned at birth. There were five studies that reported missing gender information [36, 43, 68, 106, 111], and four studies [71, 90, 120, 121] did not report any information on the percentage of males and females or gender distribution. The average age of the participants ranged from M = 11.09 [121] to M = 17 [106], while three studies did not report age range or mean of the sample [47, 86, 125].

### Design

All the studies included were non-experimental, with 91.4% being cross-sectional in nature (*n* = 85). These studies employed surveys to investigate the impact of family-related psychosocial factors on suicidal ideation among adolescents. Among the rest of the studies, six were longitudinal study designs [47, 57, 59, 62, 88, 102]. One was a randomized controlled trial [70], and another utilized a cohort study design [121]. The longitudinal and trial-based studies were given greater weight due to their stronger methodological designs and better ability to provide temporal evidence.

### Quality assessment

The quality assessment result of the included studies is included in the supplementary material 2. All the studies were included and were of acceptable overall quality. Across the included studies, items related to identifying confounding factors and handling those factors were mostly rated "no" by the reviewers, indicating that potential confounders were either not clearly specified or not adequately addressed in the analysis.

### Findings

Based on the narrative synthesis of the included studies, a total of eight domains of family-related psychosocial factors were identified (see Table 3 in Supplementary Material 3): hostility inside the family, family communication, attachment with family members, parental emotional availability, parental control, parental psychopathology, parental socioeconomic and educational status, and parental substance use. Within each domain, the direction of effect and statistical significance are summarized in Supplementary Material 1. Additionally, Table 4 (see Supplementary Material 3) lists all variables, along with the total number of times they were repeated across all included studies, for each domain. This section sheds light on the understanding of family contexts that either protect or aggravate the risk of suicidal ideation among adolescents.

**Table 3:** Outcome

Sl No	Domains	Citations
1	Hostility inside the family	[37, 48, 50, 65, 67, 71, 74, 76, 81, 84, 86, 87, 94, 99, 103, 105, 106, 121, 122, 124, 128]
2	Family Communication	[39, 43, 45, 58, 60, 63, 69, 70, 77, 79, 84, 87, 88, 104, 105, 107, 110, 113, 116–119, 127]
3	Attachment with family members	[38, 45, 57, 58, 69, 77, 79, 84, 87, 88, 96, 97, 101, 107, 113, 116, 119, 122, 122, 129]
4	Parental emotional availability	[36–38, 40, 41, 47, 49, 51–55, 58, 61, 62, 64, 67, 68, 72, 76, 80–82, 85, 89, 90, 92, 93, 95, 98, 102, 106, 108, 109, 111, 112, 114, 122, 123]
5	Parental control	[35, 38, 42, 46, 49, 53, 59, 66, 68, 70, 73, 76, 97, 102, 111, 114, 127]
6	Parental psychopathology	[55, 56, 61, 75, 121, 124]
7	Parental socioeconomic and educational status	[44, 45, 78, 95, 100, 114, 116, 119, 120, 126, 128]
8	Parental substance use	[54, 98, 115]

**Table 4:** Outcomes of the studies included

Category	Outcome	Studies
Hostility inside the family	Parent–child conflict (1), Family conflict (4), Parent-adolescent conflict (3), Parental conflict (1), Mother-adolescent conflict (1), Interparental conflict (1), Conflictual independence (1)	[37, 50, 74, 81, 84, 86, 91, 94, 103, 122, 128]
	Family dysfunction (2)	[65, 67]
	Parental abuse (2), Physical abuse (2), Psychological abuse (1), Parental hostile behavior (1), Family violence (1), Parental neglect (1), Sibling bullying (1), Rejection at home (1), Family incivility (1)	[48, 67, 76, 87, 99, 105, 121, 124]
	Negative feelings towards family (1)	[106]
Family Communication	Family communication (3), Parent-adolescent communication (2)	[39, 63, 110, 118, 125]
	Family functioning (8)	[60, 70, 83, 103–105, 110, 117]
Attachment with family members	Satisfaction with family (1), subjective satisfaction with relationships among family members (1), perceived parents' dissatisfaction (1)	[113, 116, 122]
	Family cohesion (3), Parental closeness (1), Family connectedness (4), Family togetherness (1)	[38, 57, 79, 88, 96, 101, 102, 119, 122]
	Parent–child attachment relationship (1), Parental attachment (5), Perceived relationship quality with parents (1), Mother-Adolescent Relationship Quality (1)	[45, 58, 69, 77, 84, 87, 97, 107]
Parental emotional availability	Perceived social support from family (4), Perceived family support (1), Family social support (3), Family support (4), Support from family (1), Parental social support (1), Perceived parental social support (1), Parental support (6), Lack of parental support (1), Supportive parents’ responses to children’s negative emotions (PRCNE) (1)	[36, 38, 40, 41, 47, 52, 58, 61, 64, 67, 72, 80, 82, 85, 90, 92, 95, 106, 108, 109, 114, 122, 123]
	Familism (1)	[37]
	Parental understanding (7), Understanding parents (1)	[49, 53–55, 68, 92, 93, 112]
	Verbal affirmation from parents (1), Perceived acceptance from mother (1), expecting too much by parents (1)	[51, 81, 98]
	Parental involvement (1), Parental academic interest (1), Checking homework by parents (1), Parents’ participation in education (1), Parental phubbing (1), Mother phubbing (1)	[51, 62, 76, 89, 98, 102]
	Parental care (2)	[102, 111]
Parental control	Parental psychological control (2), Parental control (1)	[35, 66, 114]
	Parental monitoring (4), Parental supervision (4), Autonomy (1)	[53, 68, 70, 73, 76, 102, 111]
	Parental invasion of privacy (1), Parental intrusion of privacy (2)	[49, 53, 68]
	Positive parenting (1), Negative parenting style (2), Harsh parenting (1), Parenting Style (2)	[38, 42, 46, 59, 97, 127]
Parental psychopathology	Parental psychopathology (1), Parental depressive mood (1), Parental depressive symptoms (1),	[56, 121, 124]
	Parental suicidal ideation(2), Family suicide history (2), Family history of suicide attempts (1)	[55, 56, 61, 75]
Parental socioeconomic and educational status	Parental marital status (3), Marital conflict (1), Family structure (3), Socio-economic status (2), Family economic deprivation (1), Family economic status (1), Family economic pressure (1), Family work status (1), Parents’ work stress (1), Family financial stress (1)	[44, 45, 78, 95, 100, 114, 116, 119, 120, 126, 128]
	Parent education (1), Family educational status (1)	[100, 120]
Parental substance use	Parental use of tobacco (1), Parental drinking (1), Parent/s smoking	[54, 98, 115]

### Hostility inside the family

Out of 94 studies, a total of eleven studies examined the impact of family conflict-related processes on adolescents' suicidal ideation. These studies included variables such as parent–child conflict, family conflict, parent-adolescent conflict, parental conflict, mother-adolescent conflict, interparental conflict, and conflictual independence. Ten studies reported a positive association between conflictual family environments and increased suicidal ideation [37, 50, 74, 81, 84, 86, 91, 94, 122, 128]. However, a study on Portuguese adolescents (*N* = 534) reported a negative relationship, which is explained through the concept of conflictual independence. This study indicated that a conflictual family environment might lead to conflictual independence from parents, ultimately lowering suicidal ideation [103]. In addition to family conflict, family dysfunction [65, 67] (imbalance in one or more domains of healthy family functioning, which includes problem solving, communication, roles, affective responsiveness, involvement, behavioral control, and general functioning) and negative feelings towards family [106] have shown a significant positive association with suicidal ideation.

In addition to a conflictual environment, experience of direct abuse from parents (physical and psychological), as well as family incivility (unconscious uncivilized behavior of family members towards adolescents by being arrogant, insensitive, impolite, or disrespectful, which causes unintentional and indirect harm), has a positive association [48, 76, 99, 105, 121, 124]. Whereas, two studies focusing on parental neglect and sibling bullying reported no significant association with suicidal ideation [67, 87].

### Family communication

Communication-related variables, such as family communication and parent-adolescent communication, were examined across five cross-sectional studies [39, 63, 110, 118, 125]. Among the three studies that analyzed family communication, two studies [39, 63], evaluated its impact on suicidal ideation indirectly by studying it as a moderator between traditional victimization and suicidal ideation, and physical abuse and suicidal ideation, respectively. In a study conducted on Spanish adolescents (*N* = 1,007), poor family communication (failure to openly discuss important issues typical of adolescents’ life) significantly increased the risk of suicidal ideation among those subjected to cyber victimization [39]. Similarly, the indirect effect of improved family communication on suicidal ideation among Spanish adolescents (*N* = 1,405) was also found to be significant, further reducing the risk of suicidal ideation [63]. Furthermore, the studies indicate a gender difference in the context, showing low suicidal ideation only among males and not among females [125]. However, parent-adolescent communication [110, 118] was shown to have a significant negative association with suicidal ideation, both between males and females.

Family functioning was also assessed across seven cross-sectional studies [60, 83, 103–105, 110, 117] and one randomized controlled trial (RCT) [70]. Family functioning refers to the family’s overall health and dynamics among its members, determined by interactions, communication, problem-solving, and stress management [105]. All the cross-sectional studies indicated a significant negative correlation of family functioning with suicidal ideation across genders. In one study on Taiwanese adolescents [104], family functioning moderated the effect of problematic cellular phone use on suicidal ideation (*N* = 5051). However, in an RCT, family functioning did not significantly predict suicidal ideation over a 12-month period [70].

### Attachment with family members

Seven cross-sectional studies [38, 79, 96, 101, 102, 119, 122] and two longitudinal studies [57, 88] have indicated that family cohesion and connectedness are negatively associated with suicidal ideation. Family cohesion and connectedness are typically measured through family cohesion, parental closeness, family connectedness, and family togetherness.

A total of three cross-sectional studies examined adolescents’ subjective satisfaction with family members and their association with suicidal ideation [113, 116, 122]. All studies consistently reported a significant positive association between suicidal ideation and low levels of satisfaction with family members. A study on Chinese adolescents [122] reported that students who perceived their parents as dissatisfied attributed it to their academic performance, thereby increasing their risk of SI.

Eight cross-sectional studies [45, 58, 69, 77, 84, 87, 97, 107] have found a significant negative association between suicidal ideation and healthy attachment in the family. Attachment in this context is studied in terms of parent–child attachment, parental attachment, perceived relationship quality with parents, and mother-adolescent relationship quality. A study on Chinese adolescents (*N* = 8680) revealed that healthy attachment has a direct effect as well as an indirect effect on low suicidal ideation risk [69]. Additionally, another study of Portuguese adolescents (*N* = 604) reported a complex result, noting that the direction of the effect varied according to the domains of parental attachment; quality of emotional bond negatively, and the individuality and exploration dimension positively impacted suicidal ideation risk [97].

### Parental emotional availability

Support from family has been exhaustively studied in this context. All twenty-two cross-sectional studies [36, 38, 40, 41, 52, 58, 61, 64, 67, 72, 80, 82, 85, 90, 92, 95, 106, 108, 109, 114, 122, 123] and one longitudinal study [47] that explored the relationship between family support and suicidal ideation have consistently shown a significant negative association.

Fifteen cross-sectional [37, 49, 51–55, 62, 68, 76, 81, 89, 92, 93, 98, 102, 111, 112] and two longitudinal studies [62, 102], have worked on a variable similar to family support, i.e., familism (prioritizing family honor, sacrifice, and dependence to support collective family well-being [37]), parental understanding, parental acceptance, parental engagement, and parental care. All variables related to parental understanding, acceptance, care, and time spent with adolescents were reported as protective factors against suicidal ideation risk, indicating a negative association between suicidal ideation and parents' emotional availability. Additionally, a longitudinal study on the impact of parental phubbing on Chinese adolescents (*N* = 2,407) indicated increased risk of suicidal ideation across three different time points [62].

### Parental control

Parental control-related in the context of adolescent suicidal ideation was studied in fourteen cross-sectional studies, which included parental psychological control, parental monitoring or supervision, parental invasion of privacy, and different parenting styles [35, 38, 42, 46, 49, 53, 59, 66, 68, 70, 73, 76, 97, 102, 111, 114, 127]. However, the association of control-related variables and suicidal ideation is not straightforward. Parental psychological control had a significant positive association with suicidal ideation [35, 66], whereas high parental physical control was negatively associated with suicidal ideation [114]. Studies on parental monitoring have reported mixed results, an RCT study over a 12-month period noted that parental monitoring (designing strategies to keep adolescents’ behavior in check and reduce their chances of defying rules) negatively predicted SI, aligning with the results of a cross-sectional study [70, 73]. However, another cross-sectional study reported a positive association of parental monitoring with suicidal ideation [68]. Parenting style was also one significant predictor in this context; negative parenting style, such as authoritarian and permissive parenting styles, positively predicted suicidal ideation [42, 46], whereas authoritative parenting reduced the risk of suicidal ideation [97, 127].

### Parental psychopathology

A small group of studies identified the impact of parental mental health and suicidal behavior on adolescent suicidal ideation. The studies included five cross-sectional and one cohort studies [55, 56, 61, 75, 121, 124], which included variables like parental psychopathology, parental depressive mood, parental depressive symptoms, parental suicidal ideation, family suicide history, and family history of suicide attempts. All the studies included indicated a positive and significant association with suicidal ideation; for instance, adolescents with parents diagnosed with depressive mood disorders had a higher risk of suicidal ideation [56]. Similarly, studies that reported the impact of parental suicidal behavior also noted a higher risk of suicidal ideation among the adolescents [55, 56, 61, 75]. This group of studies indicates the importance of early diagnosis and treatment of parental mental health issues, and active work towards the reduction of parental suicidal behaviors.

### Parental socioeconomic and educational status

A total of eleven cross-sectional studies identified variables related to economic and marital status, which included parental marital status, marital conflict, family structure, socio-economic status, family economic deprivation, family economic status, family economic pressure, family work status, parents’ work stress, family financial stress, parent education, and family educational status [44, 45, 78, 95, 100, 114, 116, 119, 120, 126, 128]. The studies focusing on the financial and work status of parents reported similar results, indicating a significant positive association with suicidal ideation. A study on Chinese adolescents (*N* = 907) indicated that parents’ work stress is positively associated with suicidal ideation; however, adolescents with mothers who had more work stress reported higher suicidal ideation risk as compared to those with fathers who had more work stress. Additionally, the marital status of parents had no significant association with suicidal ideation [114, 126], whereas one study noted that parental marital status increased the risk of suicidal ideation [128].

### Parental substance use

A limited number of cross-sectional studies examined the impact of parental substance use on adolescent suicidal ideation, which included the variables parental use of tobacco, parental drinking, and parent/s smoking [54, 98, 115]. Two studies reported a significant and positive association with suicidal ideation, especially the study on parental drinking, which was reported to be the only significant predictor of adolescent suicidal ideation among other studied family-related variables [98]. Similarly, parents who used tobacco were reported to have adolescents with an increased risk of suicidal ideation [54]. Whereas, parent/s' smoking did not report a significant impact on adolescent suicidal ideation [115]. Therefore, although the evidence remains limited, the findings contribute to the complex understanding of adolescent suicidal ideation attributed to parental substance use, indicating a need for further investigation.

## Discussion

This review aimed to understand the impact of family-related psychosocial factors on adolescent suicidal ideation. It provided an understanding of different domains of family-related psychosocial factors, ranging from adverse family environments elevating suicidal ideation risks to a supportive family environment being protective against it. The identified outcomes from the narrative synthesis were categorized into eight domains. The findings reported the direction of effect and statistical association among the factors, which varied across the identified domains. The pattern could be better understood in terms of risk and protective factors based on their impact; therefore, the reported outcomes were further classified into these categories. This pattern provided a better understanding of adolescent suicidal ideation, highlighting the dynamic influence of family-related psychosocial factors.

### Risk factors

Based on the narrative synthesis, five domains were identified as risk factors of suicidal ideation among students, i.e., hostility inside the family, parental psychopathology, parental substance use, parental control, and parental socioeconomic and educational status.

Parental conflictual [37, 50, 65, 67, 74, 81, 84, 86, 91, 94, 122, 128] and abusive behaviors [48, 76, 87, 99, 105, 121, 124] towards adolescents is consistently associated with suicidal ideation, echoing the existing literature [129–131]. Furthermore, exposure to such negative experiences results in adolescents’ negative feelings towards their family, eventually leading to increased suicidal ideation risk [106]. On the other hand, one study reported no significant association between parental neglect and suicidal ideation [67], which might indicate that parental neglect alone cannot predict suicidal ideation and behaviors, aligning with a longitudinal study [132]. A conflictual family environment might lead to conflictual independence, where psychological separation from parents may lead to liberation from negative emotions such as anger, guilt, mistrust, and resentment [103]. More empirical studies should investigate whether conflictual independence can have a protective impact.

Parental psychopathology, manifesting either as poor parental mental health or their suicidal behavior [55, 56, 61, 75, 121, 124] was a risk factor for adolescent suicidal ideation. This finding aligns with a recent review that reported that parental psychopathology poses a threefold higher risk of suicidal ideation as compared to other risk factors [133]. However, of the identified studies extracted for review, only a small number addressed the variable, indicating a need to explore it in depth and develop interventions targeting early diagnosis, designing better treatment plans, and reducing the risk of familial transmission of risky behaviors [133].

Similarly, parental substance use (tobacco [54] and alcohol use [98]) aggravated the risk of suicidal ideation among adolescents. In terms of tobacco use, however, contradictory findings exist [115], with some studies showing an indirect association through loneliness [134]. Therefore, this review positions parental substance use as an important but understudied family-related psychosocial factor. It highlights the need to develop interventions that can simultaneously aim at reducing parental substance use and its impact on suicidal risk.

In contrast to the other domains, which are relatively risky, evidence on parental control yielded mixed results. Across the identified studies, types of parental control, i.e., psychological and physical control, showed high and low suicidal ideation risk, respectively. At the same time, the findings on parental monitoring did not report uniform results. This discrepancy in the pattern may reflect differences between autonomy-based and surveillance-based monitoring, which are often used under the same category in quantitative studies, thereby overlooking the impact of these differences on suicidal ideation [135]. Whereas the findings on parenting styles align well with the existing broader literature [136]. The identified studies in the review failed to highlight the impact of cultural factors on parenting styles and adolescent suicidal ideation. Therefore, the findings provide evidence that parental control and its impact on suicidal ideation vary based on their types and the adolescents’ experiences. Additionally, it underscores the need to use accurate measures of parental control and to interpret results accordingly, accounting for relevant cultural differences.

Similar to the parental control domain, parental socioeconomic and educational status also yielded mixed results. The included studies identified under the dimension predominantly reported a significant positive association of poor socioeconomic, financial, and work status with suicidal ideation. However, the review noted that the association between parental work stress and adolescent suicidal ideation was gender specific, i.e., the risk of suicidal ideation increased when mothers face more work stress as compared to fathers. This association may be attributed to the mother being the primary caregiver, providing emotional care, warmth, and support, a disruption in which, due to occupational severity, may increase the risk of suicidal ideation [95]. Additionally, the findings on the association of parental marital status with increased suicidal ideation persist; however, contradictory evidence exists indicating no significant association [114, 126, 128]. This discrepancy may result from differences in measurement scales, unidentified moderators that may impact the study. Therefore, the inconsistency could be addressed by using multiple assessment scales, employing other study designs, such as longitudinal or intervention-based designs, and conducting moderation analyses.

### Protective factors

Among the other domains synthesized in the review, family communication, attachment with family members, and parental emotional availability were the protective family-related factors that reduced the risk of suicidal ideation among adolescents.

Positive family communication deterred suicidal ideation [110, 118, 125], sometimes through a mediational variable such as the reduced impact of cybervictimization [39, 63, 137] on suicidal ideation. This review noted that healthy and open communication of adolescents with their family members significantly reduced the risk of suicidal ideation. It also highlighted that adolescents with low levels of family communication reported a high risk of suicidal ideation due to being subjected to cyber victimization, but the association was insignificant among those with healthy family communication.

In addition, the cohesion-related and family functioning variables were also noted to be protective factors reducing suicidal ideation. This pattern is supported by a review based on 12 cohort studies that provided evidence on adolescents’ low suicidal ideation risk being attributed to healthy family functioning [138]. Nonetheless, an RCT study of family functioning did not predict suicidal ideation over a 12-month period [70], contrasting with the cross-sectional pattern observed in other studies focusing on family functioning. This discrepancy may be attributed to methodological differences between RCTs, which tend to use an intervention-based approach [139]. At the same time, the existing evidence on the protective impact on suicidal ideation makes it reliable to target family cohesion, functioning, and open communication in designing prevention strategies against suicidal ideation.

Positive attachment with family members, in terms of emotional availability and support, significantly buffered adolescents’ initiation or escalation of suicidal ideation. However, given that the data are predominantly cross-sectional, evidence for causal risk reduction is limited, further underscoring the need for intervention-based studies. A study exploring the domains of parental attachment, for instance, noted differences in the impact of dimension-based parental attachment on suicidal ideation; however, the causal evaluation of dimensional differences is limited due to the cross-sectional nature of the study [97]. Therefore, to understand the nature of the impact of different domains of family-related variables, longitudinal or intervention-based studies are required. It may help develop strategies aimed at improving parent–child relations, eventually developing effective suicidal prevention plans [140].

Further, the implication at broader-level societal efforts may also protect adolescents against suicidal ideation before it reaches crisis situations requiring medical attention. Policies such as public awareness campaigns, school-based mental health education programs, and parent-focused communication activities that encourage parents to be more open, non-judgmental, and emotionally supportive while communicating would help provide effective protection. Such prevention approaches may also prove beneficial in situations with limited mental health resources [141].

### Strengths and limitations

This review is the first to explore the impact of diverse domains of family-related psychosocial factors on adolescent suicidal ideation holistically. It includes a range of domains, including family environment, parental mental health, communication, family structure, parent–child attachment, parental control, and substance use. It identifies actionable targets such as healthy family communication, emotionally available parents, authoritative parenting, and positive parental attachment for designing targeted suicide prevention strategies. Additionally, it also identifies problem sources that, if addressed, may reduce suicidal ideation risk, for instance, early diagnosis of parental mental health issues, practicing work-life balance, encouraging autonomy-based monitoring of children, and disruption of violence-based parenting practices may significantly reduce the risk of adolescent suicidal ideation. The review follows SWiM guidelines to report and discuss the findings of included studies [34]. It uses narrative synthesis to report the heterogeneity of the results and to address the limitations of the included studies.

This review is limited to peer-reviewed articles identified through a systematic search, which may have overlooked some relevant studies due to the unavailability of open-access articles or those available only in print. The review included only quantitative studies, failing to capture the mechanisms and lived experiences typical of qualitative research [142]. It focuses on adolescents, excluding other age groups, where the influence of family-related psychosocial factors may differ developmentally. The studies included in the review are predominantly cross-sectional, limiting causal inference; therefore, future studies could focus on intervention-based or longitudinal designs to infer causality [143]. The review also included studies mainly focusing on male and female genders, with only two studies addressing the LGBTQ + community, thereby limiting the understanding of suicidal ideation across several gender identities. Based on the country-wise presentation, limited studies from India and other South Asian countries limit generalizability to the understanding of adolescent suicidal ideation in these regions, underscoring the need to focus on adolescents in South Asia. Finally, a meta-analysis of the included studies was not possible due to the heterogeneous nature of the findings; therefore, it relied on the vote-counting method to report direction of effect and statistical associations.

## Conclusion

This review provides valuable insights into the diverse domains of family-related psychosocial factors and their bidirectional influence on suicidal ideation. While the identified domains provide valuable information on several factors and their influences, there is still a dearth of evidence exploring the impact of certain critical factors on suicidal ideation in-depth. For instance, the impact of parental psychopathology, physically handicapped parents, different sexual identities of adolescents, parental substance use, and gender-based differences in the occupational status of parents should be further explored. A deeper exploration would help design interventions prioritizing gender-informed approaches, culture-appropriate training skills in mental health care professionals, and provide family-centered suicide prevention strategies.

Furthermore, in developing prevention strategies, practices such as strengthening parent–child relationships, encouraging open communication between parents and children, and supporting mental health in both parents and adolescents could be considered. The findings of this review are hoped to help and guide practitioners, mental health specialists, and suicide prevention activists in developing family-centered prevention programs, encouraging healthy family relations, and promoting mental health well-being among adolescents.

## Supplementary Information

Supplementary Material 1.

Supplementary Material 2.

Supplementary Material 3.

## Data Availability

All data and materials supporting the findings of this study are available within the paper and its Supplementary Files. The data extracted from the retrieved studies are provided in Supplementary file 1, and the quality assessment data are provided in Supplementary file 2. Additionally, all the tables included in the study were uploaded separately in the system, and attached in Supplementary file 3.
