# Supplementary material for: The impact of family-related psychosocial factors on suicidal ideation among adolescents: a systematic review
Source: BMC Public Health. 2026 Jul 9;26:2743. doi: 10.1186/s12889-026-27928-7 (PMC13632444; doi:10.1186/s12889-026-27928-7)
Supplement: Supplementary file 1 [file 12889_2026_27928_MOESM1_ESM.pdf]

Author, (Year), Location	Sample Size	Gender	Age (Mean, SD)	Study Design	Family-related psychosocial factor		Measures	Main Result (Suicidal Ideation - SI) (p: level of significance, OR: odds ratio, RR: Risk ratio, HR: hazard ratio, M: mean, SD: standard deviation, β: beta coefficient, SE: standard error)	
					Risk Factor/s	Protective Factor/s		Risk Factor/s	Protective Factor/s
Picou et al., (2024), USA	N = 250	1) 98 males (39.2%); 2) 152 females (60.8%)	12-17 years (M = 14.84, SD = 1.48)	cross-sectional survey	Parent-child conflict	Familism	1) Conflict Behavior Questionnaire (Foster & Robin, 1989); 2) Attitudinal Familism Scale (Steidal & Contreras, 2003); 3) Modified Scale for Suicide Ideation (MSSI; Miller et al., 1986)	1) Suicidal ideation demonstrated significant, positive associations with youth's ratings of parent-child conflict (r = 0.26, p<0.01); 2) higher levels of familism were associated with lower levels of self-reported suicidal ideation (r = -0.32, p<0.01); 3) parent-child conflict on suicidal ideation through thwarted interpersonal needs was significant [F (3,246) = 54.41, p < .01] and accounted for 38.99% of the variance; 4) total effect of parent-child conflict on suicidal ideation was positively significant (point estimate = 0.59, SE = 0.14, CI [0.32, 0.87]); 5) both indirect effects were significant, perceived burdensomeness demonstrated a stronger indirect effect with parent-child conflict and suicidal ideation than thwarted belongingness.	1) Familism significantly moderated the indirect effect of parent-child conflict on suicidal ideation through perceived burdensomeness (index of moderated mediation = 0.006, SE=0. 0022, 95% CI [0.0019, 0.0104], p < .01) and thwarted belongingness (index = 0.0016, SE=0. 0009, 95% CI [0.0001, 0.0036], p < .05); 2) familism significantly moderated the indirect relationship of thwarted interpersonal needs on parent-child conflict and suicidal ideation.
Keshky, (2024), Saudi Arabia	N = 614	1) 239 males (38.9%); 2) 375 females (61.1%)	12-18 years (M = 16.16, SD = 1.88)	cross-sectional survey	-	1) Support from family; 2) Positive parenting; 3)family cohesion; 4) family adaptability	1) Multidimensional Scale of Perceived Social Support (Zimet et al., 1988); 2) Positive parenting was measured by the Alabama Parenting Questionnaire (APQ; Frick, 1991); 3) The Family Adaptation and Cohesion Evaluation Scale (FACES III) (Olson et al., 1985); 4) The Positive and Negative Suicide Ideation (PANSI) Inventory (Osman et al., 2016)		1) The protective factors that were strongly connected to negative suicide ideation were support from family, family cohesion, and family adaptability, while protective factors connected to positive suicide ideation were positive parenting and family adaptability; 2) the most central protective factor node with regard to expected influence was family cohesion; 3)The most central protective factor node with respect to strength was family cohesion.
Buelga, (2024), Spain	N = 1007	1) 523 males (51.9%); 2) 484 females (48.1%)	12-18 years (M = 14.73, SD = 1.75)	cross-sectional survey	-	Family Communication	1) Parent-Adolescent Communication Scale (Barnes & Olson, 1982; adaptation by Estévez et al., 2005); 2) Suicidal Ideation Scale (Mariño et al., 1993; adaptation by Sánchez-Sosa et al., 2010).		1) family communication is a significant moderating variable of the relationship between traditional victimization and cybervictimization (B = -.09, p < .05); 2) there is a positive relationship between traditional victimization and cyber-victimization in both low and high levels of family communication (B = .52, SE = .02; 95% CI: 0.481, 0.560: low level of communication; B =. 42, SE = .03; 95% CI: 0.371, 0.471: high level of communication), however the relationship is more pronounced for low levels of family communication; 3) The result indicated a stronger relationship between traditional victimization and cyber-victimization in those adolescents with low levels of family communication; 4) Cyber-victimization is positively related to suicidal ideation in those adolescents who have low levels of family communication (B = .34, SE = .07; 95% CI: 0.204, 0.473). However, this relationship is not significant in adolescents with high levels of family communication (B =.10, SE =.09; 95% CI: -0.073, 0.285); 5) no moderating effect of family communication is observed in the relationship between traditional victimization and suicidal ideation (B = .08, SE =.07; 95 % CI: - 0.060, 0.220);
Ding et al., (2022), China	N = 4574 families	1) 2534 males (55.4%); 2) 2040 females (44.6%)	M = 16.28, SD = 1.09	cross-sectional survey	Parents' non-supportive responses (PRCNE): punitive response and parental distress	1) Supportive parents' responses to children's negative emotions (PRCNE): emotion-focused response, problem-focused response, and expression encouragement; 2) Perceived relationship quality with parents	1) Coping with Children's Negative Emotions Scale-Adolescent Version (CCNES-AV, Fabes and Eisenberg, 1998; Fabes et al., 2002); 2) Perceived relationship quality with parents (scale by Raja et al., 1994); 3) for SI, one question was asked: During the past 12 months, did you ever seriously consider attempting suicide?	1) Perceived relationship quality with parents had a significant indirect effect between parents' non-supportive responses and SI (p<0.05); 2) parents' non-supportive responses may alter adolescents' trust towards their parents and perceived proximity, which may increase their chances of SI.	1) Adolescents' perceived relationship quality with parents was negatively assciated their SI (r = -0.184; p<0.001); 2) perceived relationship quality with parents had a significant indirect effect between parents' supportive responses and SI (p<0.05); 3) parents' supportive responses may alter adolescents' trust towards their parents and perceived proximity, which may buffer against SI.

Sun et al., (2022), China	N = 371	1) 183 males (49.3%);<br>2) 188 females (50.7%)	12-16 years (M = 13.98, SD = 0.88)	Longitudinal	Harsh Parenting		1) Harsh parenting was measured using eight items developed by Simons et al. (1991) and validated by Wang (2017);<br>2) Beck Scale for Suicide Ideation (BSI).	1) Harsh parenting and SI were positively correlated (r = 0.22**, p<0.01); 2) harsh parenting positively predictced SI (β= 0.30, p < 0.001); 3) harsh parenting also had a statistically indirect effect on SI (indirect effect = 0.06; Boot SE = 0.02; 95% CI = [0.029, 0.110]); 4) With mindfulness as moderator, relationship between harsh parenting and SI was significantly associated (β= 0.13, p<0. 05); 5) the relationship between harsh parenting and SI was stronger and postive with low mindfulness (B= 0.09, SE=0.07,t= 1.21,p>0.05); 6) With low mindfulness as moderater and depressive symptoms as mediator in the same model, the indirect effect of harsh parenting on SI was strong and significant (indirect effect = 0.04,; 95% CI [(0.001, 0.066]), but insignificant when mindfulness was low (indirect effect = 0.03; CI = [(-0.001, 0.065]).	
Lee et al., (2021), South Korea	N = 7333	1) 3608 males (49.2%);<br>2) 3725 females (50.8%)	12-19 years (M = 15.39, SD = 1.70)	Cross-sectional survey	1) Parental abuse;<br>2) Parental neglect;<br>3) Family dysfunction	Parental support	1) SI: "Have you ever thought about suicide in the past 12 months?";<br>2) Parental abuse: two items inquiring the extent of parents' physical and verbal abuse in the past 12 months;<br>3) Parental neglect: Korean version of the Parenting Behavior Inventory (PBI);<br>4) Family dysfunction: "In the past 12 months, I have experienced high levels of stress as my family was dysfunctional";<br>5) Parental support: four items asking about the level of parents' respect and care for their child's autonomy.	1) Parental abuse (OR = 1.22, p<0.001) and family dysfunction (OR = 1.33, p<0.001) had a significant positive association with SI;<br>2) Parental abuse and family dysfunction were significant risk factors, but parental neglect did not have any association with SI;<br>3) Parental neglect may result in cognitive and psychological consequences, however it is not directly related to SI.	1) Parental support had a significant negative association with SI (OR = 0.96, p<0.01);<br>2) parental support for autonomy had a protective effect against SI among adolescents.
Handley et al., (2019), USA	N =164	1) 0 males (0%);<br>2) 164 females (100%)	M = 14 years, SD = 0.85	Cross-sectional survey	1) Adolescent Maltreatment;<br>2) Mother-Adolescent Conflict;<br>3) Mother-Adolescent Relationship Quality.		1) Childhood Trauma Questionnaire (CTQ; Bernstein et al., 2003);<br>2) Conflict Behavior Questionnaire (CBQ-20; Robin &Foster, 1989);<br>3) Parent and Peer Attachment (IPPA; Armsden & Greenberg, 1987);<br>4) Passive or Active Suicide Ideation:<br>Indicator 1: "Recurrent thoughts of death" (current) from KSADS (Kaufman et al., 1997),<br>Indicator #2: "Suicidal ideation" (current) from KSADS (Kaufman et al., 1997),<br>Indicator #3:"I wish I were dead" was from the BDI-Y (Beck et al., 2005).	1) higher levels of child maltreatment were associated with greater SI based on all three indicators (rs = .32–.48, ps < .001);<br>2) Poor relationship quality and conflict were associated with greater adolescent SI (relationship quality: rs = .28 to .41, p < .001; conflict: rs = .23–.28, ps < .001);<br>3) 22.1% of nonmaltreated depressed adolescents and 48.8% of maltreated depressed adolescents reported recurrent thoughts of death (v2 (1) = 12.31, p < .001);<br>4) 11.7% of nonmaltreated depressed adolescents indicated suicide ideation, compared to 26.8% of maltreated depressed adolescents (v2 (1) = 5.80, p = .02);<br>5) mother–adolescent conflict and worse mother–adolescent relationship quality individually predicted greater suicide ideation (bconflict = .18, p < .01, bquality = .28, p < .01).	
Lardier et al. (2016), USA	N = 488	1) 232 males (47.5%);<br>2) 256 females (52.5%)	10-18 years (M = 13.81, SD = 1.49)	Cross-sectional survey	Family conflict		1) Student Survey of Risk and Protective Factors/Family Conflict Scale (Gorman-Smith et al., 1996);<br>2) Youth Risk Behavioural Surveillance Survey (Kann et al., 2014)	1) SI was positively correlated with family conflict (r = 0.373, p<0.01);<br>2) using logistic regression, family conflict increased SI risk among males (OR = 1.25, p<0.01) and females (OR = 1.16, P<0.01) by 25% and 16% respectively;<br>3) family conflict had a significant indirect effect on SI as a mediator both in males (b = 0.087, p = 0.05, 95% CI [0.017, 0.194]; (b = 0.041, p = 0.017, 95% CI [0.016, 0.081]), and in females (b = 0.053, p = 0.03, 95% CI [0.004, 0.116], (b = 0.04, p = 0.02, 95% CI [0.017, 0.078]).	

Kwok et al., (2015), China	N = 527	1) 276 males (52.4%); 2) 251 females (47.6%)	12-17 years (M = 14)	Cross-sectional survey	Physical abuse		1) Chinese version of the Parent–Child Conflict Tactics Scale (CTSPC) (Chan, 2011); 2) Suicidal Ideation Sub-scale (C-SIS) by Tse and Bagley (2002).	1) Physical abuse was found to have a significant positive association with SI in both males and females; 2) empathy (β = .08, t = 1.78, p = .05) for rational problem-solving (β = −.08, t = −2.00, p < .05) had a significant moderating effect between physical abuse and SI; 3) pshysically abused participants with high scores on rational problem solving reported low SI risk; 4) participants who were not physically abused scored low on SI, they were also found to have scored high on empathy; 5) empathy and rational problem-solving also significantly moderated the relationship between physical abuse and SI in females (β = .28, t = 4.46, p < .01), but not in males (β = .32, t = 5.47, p = . 001).	
Kwok et al., (2013), China	N = 560	1) 296 males (52.9%); 2) 264 females (47.1%)	12-17 years (M = 14)	Cross-sectional survey	1) Physical abuse; 2) Psychological abuse	Family functioning	1) Chinese version of the Parent–Child Conflict Tactics Scale (CTSPC; Chan, 2011) - physical abuse and psychological abuse; 2) Chinese Family Assessment Instrument (C-FAI; Shek, 2002) - family functioning; 3) Suicidal Ideation Sub-scale (C-SIS) by Tse and Bagley (2002).	1) Physical abuse was significantly correlated with high SI risk (r = .22, p < .001); 2)	1) Perceived family functioning was negatively correlated with lower SI (r = -0.47, p<0.01); 2) high mutuality (β = 0.07, t = 2.51, p <0.05) and high family communication (β = 0.09, t = 3.59, p < .01) significantly moderated the association between physical abuse and SI, with high mutuality and family communication lowering SI risk.
Samm et al., (2009), Estonia	N = 4389	1) 2178 males (49.6%); 2) 2211 females (50.4%); i) 1404 (683 males (48.6%); 721 females (51.4%)) - 11 years; ii) 1434 (712 males (49.7%); 722 females (50.3%)) - 13 years; iii) 1544 (779 males (50.5%); 765 females (49.5%)) - 15 years	11 years (M = 11.8, SD = 0.3); 13 years (M = 13.8, SD = 0.4); 15 years (M = 15.8, SD = 0.2)	Cross-sectional survey	1) family economic deprivation	1) subjective satisfaction with relationships among family members; 2) perceived ease of talking about worries to family members; 3) family structure;	1) 'How satisfied are you with the relationships amongst members of your family?' - satisfaction with relationships; 2) 'How easy is it for you to talk about your worries to the following people: mother, father, stepmother, stepfather, elder sister, elder brother, grandmother and grandfather?' - communication quality; 3) included questions about the respondent's permanent home (or the one they live in most of the time) and the other people living there (parents, step-parents, grandparents or others) - family stucture; 4) Family Affluence Scale (FAS) - family deprivation; 5) The 11-year-olds were asked: 'Over the past 12 months, have you ever had a serious thought that it would be better if you weren't alive any more?' The 13 and 15-year-olds were asked: 'Over the past 12 months, have you ever had a serious thought of killing yourself (suicide)?' - SI.	1) boys with economic deprivation in the family had increased SI risk at age 11 (OR =1.2 and CI = 1.01–1.49); 2) girls with economic deprivation in the family had high SI risk at age 11 (OR = 1.2 and CI = 1.02–1.46) and 15 (OR = 1.5 and CI = 1.20-1.76).	1) higher satisfaction in relationships with family members among boys was consistently found to be associated with lower SI risk across all age groups (OR = 0.7 and CI = 0.62–0.76 at age 11 years; OR = 0.7 and CI = 0.62–0.78 at age 13 years; and OR = 0.7and CI = 0.61–0.96 at age 15 years for boys; and OR = 0.8 and CI = 0.69–0.83 at age 11; OR =0.7 and CI = 0.61–0.76 at age 13; and OR = 0.7 and CI = 0.63–0.76 at age 15 years for girls); 2) for family communication among both boys and girls, easier communication with both mother and father were linked to low SI risk; 3) easier communication with elder sister and brother was associated with low SI risk at age 11 and 15; 4) good communication with grandparents was protective against SI risk at age 11 only; 5) as for family structure, presence of step-parents at house was linked to increased SI risk at ages 11 and 13 as compared to boys living with biological parents and for girls presence of step-parent at house was linked to high SI risk across all ages; 6) living with single parent was linked to high SI risk for girls at 15 as compared to those boys who lived with both parents; 7) good communication with elder sister among girls was linked to low SI risk at age 11 years, and at age15 with elder brothers; 8) ease of communication with grandparents was linked to low SI risk at ages 11 and 15.

Kwok & Shek, (2011), China	N = 5557	1) 2951 males (53.1%);<br>2) 2606 females (46.9%)	11-18 years (M = 13.87, SD = 1.47)	Cross-sectional survey		1) Parent-adolescent communication;<br>2) Family functioning	1) Father-Adolescent Communication Scale (FACS) and Mother-Adolescent Communication Scale (MACS) developed by Shek et al. (2006);<br>2) Chinese Family Assessment Instrument (C-FAI) by Shek, 2000;<br>3) Suicidal Ideation Sub Scale (C-SIS) of the Suicidal Risk Scale for Hong Kong students developed by Tse (2002).		1) father-adolescent communication ($r = -0.33$, $p<0.001$), mother-adolescent communication ($r = -0.42$, $p<0.001$), and family functioning ($r = -0.43$, $p<0.001$) had significant negative associations with male and female SI;<br>2) examing father-adolescent communication ($\beta = -0.13$, $p < 0.001$) and mother-adolescent communication ($\beta = -0.23$, $p < 0.001$) using hierarchical regression negatively predicted SI and explained a variance of 22%;<br>3) mother-son communication ($t = 8.54$, $p < 0.001$) and mother-daughter communication ($t = 4.69$, $p < 0.001$) had stronger association with SI as compared to father-son or father-daughter communication;<br>4) the subcales of family functioning: mutuality ($r = -0.39$, $p< 0.0083$), communication ($r = -0.36$, $p< 0.0083$), conflict and harmony ($r = -0.44$, $p< 0.0083$), parental concern ($r = -0.39$, $p< 0.0083$), parental control ($r = 0.39$, $p< 0.0083$) had significant negative correlation with SI;<br>5) using hierarchical regression analysis, it was reported that among the five subcales, except communication all four predictors significantly predicted SI, additionally family functioning explained a variance of 5% in the total sample;<br>6) a further comparison of regression coefficients revealed that family functioning ($t = 6.06$, $p < 0.001$) and mother-adolescent communication ($t = 7.96$, $p < 0.001$) had strong predicting powers on SI as compared to father-adolescent communication.
Mahfoud et al., (2010), Lebanon	N = 5109	1) 2333 males (45.7%);<br>2) 2776 females (54.3%)	13-15 years (M = 13.8)	Cross-sectional survey		Parental Understanding	1) how often in the past 30 days parents understood the adolescent's problems or worries - parental understanding;<br>2) "during the past 12months have you ever seriously considered attempting suicide?" - SI		1) using bivariate logistic regression, lack of parental understanding was found to be positively associated with SI (OR = 2.32, 95% CI [1.99, 2.70]);<br>2) with multivariate logistic regression, lack of parental understanding had a 89% increased chance at the odds of SI.
An et al., (2010), South Korea	N = 2965	1) 1514 males (51.1%);<br>2) 1451 females (48.9%)	15-18 years (M = 16.2)	Cross-sectional survey		Satisfaction with family	1) Satisfaction with the family was assessed by the following question, rated on a scale from 1 (very satisfied) to (very dissatisfied): "Choose how satisfied you are about each item below.";<br>2) "During the past year, have you ever thought you wanted to commit suicide?" - SI		1) Adolescents who had dissatisfaction with family and parents had higher chances of SI ($p<0.001$);<br>2) adolescents had higher chances of SI with parents' history of SI ($p<0.001$ for both parents) and dissatisfaction of parents with their families ($p = 0.002$, $p<0.001$);<br>3) based on logistic regression, dissatisfaction with family and parents (OR = 5.051, $p<0.001$), mother's insifficient sleep (OR = 1.417, $p = 0.020$) and mothers' history of SI (OR = 1.951, $p<0.001$) showed statistically significant odds-ratio of SI.
Logan et al., (2011), USA	N = 2598	1) 1270 males (48.9%);<br>2) 1314 females (50.6%);<br>3) 14 unknown (0.5%)	12-16+ years	Cross-sectional survey		1) Parental care;<br>2) Parental supervision	1) suicide ideation was dichotomized: (did or did not) seriously consider attempting suicide within the 12 months before the survey;<br>2) Youths were considered to have parental care if they believe their parents rewarded their good behavior by either saying something nice, giving a hug, kiss, or pat on the back, giving a reward or a special privilege, or doing a special activity with them. Youths were considered to have parental supervision if they reported that their parents either set a time for them to be home, almost always required them to leave a note or provide a phone call to state where they were going, almost always inquired to know who they were with when they were not at home, almost always knew what their activities were outside the home, or almost always knew when they would return home if they were out.<br>3) following items: "I have my parents that I can talk to and who care about my feelings and what happens to me"; "I have parents who give good suggestions and advice about my problems"; "I have parents who help me with practical problems, like to get somewhere, or help me with a job or project."		1) Both parental supervision and parental care were negatively associated with SI (OR = 0.64, $p<0.01$); 2) negative association between SI and parental care with supervision was stronger for females based on interaction term (OR = 0.56, $p<0.05$).

Springer et al., (2005), USA	N = 930	1) 446 males (48%);<br>2) 484 females (52%)	12-19 years (M = 15)	Cross-sectional survey		Perceived parental social support	1) Multidimensional Scale of Perceived Social Support (Zimet, Powell, Farley, Werkman, & Berkhoff, 1990);<br>2) having seriously considered attempting suicide in the past 12 months - SI		1) Perceived parental social support had the strongest negative association with male SI [AOR = 5.72, 95% Confidence Interval (CI) = 2.45 13.38], and also was protective against female SI.
Lee et al., (2006), China	Study 1: N = 327;<br>Study 2: N = 371	Study 1:<br>1) 0 males (0%);<br>2) 327 females (100%);<br>Study 2:<br>1) 189 males (50.9%);<br>2) 182 females (49.1%)	Study 1: 13-18 years (M = 14.39, SD = 0.68);<br>Study 2: 14 -20 years (M = 16.34, SD = 1.22)	Study 1: Cross-sectional survey;<br>Study 2: Cross-sectional survey	Study 1: Perceived parents' dissatisfaction;<br>Study 2: Parent-adolescent conflict	Study 2:<br>1) Family cohesion;<br>2) Family support	Study 1:<br>1) a scale was constructed to measure adolescents' thoughts about parents' perceived ideal child, and their perceived real child - Perceived parents' dissatisfaction;<br>2) Scale of Suicide Ideation (SSI; Beck et al., 1979).<br>Study 2:	Study 1:<br>1) perceived parental dissatisfaction in terms of academic attributes was significantly associated with adolescent SI ($r = 0.26$, $p < 0.01$);<br>2) perceived high expectations of Chinese parents led adolescents to believe that they had failed their parents and eventually increased SI.<br>Study 2:<br>1) parent-adolescent conflict had a significant positive association with SI ($r = 0.39$, $p < 0.01$);<br>2) adolescents living in a family with high parent-adolescent conflict had increased SI risk.	Study 2:<br>1) family cohesion ($r = -0.49$, $p < 0.01$) and family support ($r = -0.48$, $p < 0.01$) had a significant negative association with SI.
Shilubane et al., (2013), South Africa	N = 591	1) 291males (49.7%);<br>2) 295 females (50.3%);<br>3) 5 Unknown ()	13-19 years (M = 17, SD = 1.91)	Cross-sectional survey	Negative feelings towards family	1) Perceived social support from family;<br>2) Positive feelings towards family	1) Suicidal ideation Questionnaire (Psychological Assessment Resources, Inc.) by Reynolds (1988);<br>2) family satisfaction scale (Carver & Jones, 1992);<br>3) multidimensional scale of perceived social support (Zimet et al., 1988).	1) Negative feelings towards family had a significant positive correlation with SI ($r = 0.18$, $p < 0.01$);<br>2) linear regression analysis revealed that the frequency of SI increased when negative feelings towards the family also increased.	1) Positive feelings towards family ($r = -0.32$, $p < 0.01$) and perceived social support from family ($r = -0.14$, $p < 0.01$) had a significant negative correlation with SI;<br>2) linear regression analysis reported an increase in SI with less positive feelings towards family and low perceived social support from family.
Yang et al., (2022), China	N = 4515	1) 2268 males (50.2%);<br>2) 2247 females (49.8%)	(M = 15.24, SD = 1.66)	Cross-sectional survey		Family functioning	1) Depressive Symptom Index-Suicidality Subscale (DSI-SS) (Joiner et al., 2002);<br>2) Family APGAR (Smilkstein, 1978; Lv et al., 1995)		1) Family functioning had a significantly negative correlation with SI ($r = -0.336$, $p < 0.001$);<br>2) in a moderated mediation model, with family functioning as the main predictor, had a significant negative direct effect on SI ($\beta = -0.34$, $p < 0.001$);<br>3) with defeat as a mediator, family functioning continued to have a significant direct negative effect on SI ($\beta = -0.20$, $p < 0.001$);<br>4) in the moderated mediation model, with meaning in life as a moderator, did not have a significant effect in the relation between family functioning and SI ($\beta = 0.02$, $p > 0.05$).
Yoder & Hoyt, (2005), USA	N = 501	1) 239 males (47.7%);<br>2) 262 females (52.3%)	12-18 years (M = 14.94, SD = 1.43)	Cross-sectional survey	1) Family economic pressure;<br>2) Parental depressive symptoms;<br>3) Parental hostile behavior;<br>4) Parental physical abuse		1) Measured using six items adapted from the scale by Conger and colleagues (1992) - family economic pressure;<br>2) Center for Epidemiologic Studies Depression scale (CESD; Radloff, 1977) - parental depressive symptoms;<br>3) items adapted from six items of a scale by McGruder et al., 1992 - parental hostile behavior;<br>4) Conflict Tactics Scale (CTS; Straus, 1979) - parental physical abuse;<br>5) adolescent suicidal ideation consisted of two items: they had seriously thought about committing suicide or made plans for committing suicide.	1) based on structural equation modeling, family economic pressure is indirectly related to adolescent SI ($\beta ie= 0.02$);<br>2) parental hostile behavior was both directly ($\beta = 0.19$) and indirectly ($\beta ie = 0.19$) associated with adolescent SI;<br>3) parental physical abuse is indirectly and positively related to SI ($\beta ie = 0.06$)	
Yang et al., (2023), China	N = 479	1) 260 males (54.3%);<br>2) 219 females (45.7%)	(M = 14.29, SD = 0.81)	Cross-sectional survey	Family financial stress	Parent-child attachment relationship	1) family financial stress questionnaire (Wang et al., 2010);<br>2) Chinese version of the parent and peer attachment (IPPA) inventory (Wang & Song, 2012);<br>3) self-rating idea of the suicidal scale (Xia et al., 2002)	1) perceived familial financial stress was positively and significantly associated with SI ($r = 0.17$, $p < 0.001$);<br>2) with depression as a mediator in the moderated mediation model, between perceived family financial stress and SI, depression significantly mediated the relationship between family financial stress and SI.	1) parent-child attachment was negatively and significantly associated with SI ($r = -0.45$, $p < 0.001$);<br>2) by using parent-child attachment as a moderator, the relationship between depression and SI was moderated, that is with healthy parent-child attchment the effect of depression on SI decreases ($\beta = 0.13$, $SE = 0.04$, $t = 3.07$, $p < 0.01$)
Peltzer & Pengpid, (2012), Thailand	N = 2758	1) 1364 males (49.5%);<br>2) 1394 females (50.5%)	12-15 years	Cross-sectional survey	Lack of parental attachment		1) Parental attachment was assessed with three items. (1) Parental or guardian supervision: "During the past 30 days, how often did your parents or guardians check to see if your homework was done"? (2) Parental or guardian connectedness: "During the past 30 days, how often did your parents or guardians understand your problems or worries?" and (3) Parental or guardian bonding "During the past 30 days, how often did your parents or guardians really know what you were doing with your free time?";<br>2) "During the past 12 months, did you ever seriously consider attempting suicide?" - SI	1) based on multivariate logistic regression, lack of parental attachment was associated with SI (medium - UOR = 2.46, 95% CI [1.32, 4.57], $p < 0.01$; high - UOR = 4.23, 95% CI [2.48, 7.22], $p < 0.001$)	

Li et al., (2016), China	N = 907	1) 429 males (47.3%);<br>2) 478 females (52.7%)	(M = 15.9, SD = 1.8)	Cross-sectional survey	Parents' work stress	Parental support	1) efforte reward imbalance (ERI) questionnaire at work - chinese version (Li et al., 2005);<br>2) Parental support was measured by one single item: "In general speaking, my parents understand me, and would like to communicate with me about my problems and worries.";<br>3) Suicidal ideation was measured by a single question,"During the last 6 months, did you ever think about suicide?"	1) both parents with high work stress were reported to show low parental support as perceived by the children (OR = 3.76);<br>2) between both parents, mothers' high work stress was strongly associated with low parental support;<br>3) low parental support in turn increased adolescent SI risk (OR = 3.08);<br>4) mothers' high stress at work also had increased SI risk on adolescents as compared to fathers' work stress.	
Lee & Choi, (2015), South Korea	N = 72435	1) 36655 males (50.6%);<br>2) 35780 females (49.4%)	13-18 years	Cross-sectional survey	1) Parent education;<br>2) Socio-economic status;<br>3) living apart from family		1) Instruments for the 2013 Online Survey of Youth Health Behavior were developed by scientific experts on the survey committee.	1) Based on logistic regression, parental education did not predict SI either in boys or in girls;<br>2) Living apart from family predicted SI only in boys (OR = 1.091, 95% CI = [1.01–1.18]) but not in girls (OR = 1.065, 95% CI = [0.98–1.16]).	
Estévez-García et al., (2023), Spain	N = 635	1) 235 males (37%);<br>2) 400 females (63%)	11-17 years (M = 14, SD = 1.36)	Cross-sectional survey	Negative parenting styles		1) Child-Parental Acceptance-Rejection/Control Questionnaire (PARQ/C; Rohner, 2005) in its Spanish adaptation by Del Barrio et al. (2014);<br>2) Paykel Suicide Scale (Paykel et al., 1974) in its Spanish version by Fonseca-Pedrero et al. (2018).	1) negative parenting behavior of father (β = .15, $p < .01$), and mother (β = 0.21, $p < .01$) was significantly associated with increased SI risk;<br>2) with non-disclosure as a mediating variable, only mothers' negative socialization style had a significant indirect effect on SI (β = .12, 95% CI = [0.05, 0.23])	
Lee et al., (2018), South Korea	N = 860	1) 400 males (46.5%);<br>2) 460 females (53.5%)		Cross-sectional survey	Family conflict		1) To measure the major stress sources, the students were asked to select the most disturbing sources of stressors among the items provided, including family conflicts, academic grades, friends, health problems, and future uncertainty;<br>2) "During the past month, have you had consistently thought of suicide or developed a specific suicide plan?" - SI.	1) Family conflict had a significant positive association with SI (β = 0.087, $p < .05$).	
Wan et al., (2022), China	N = 11831	1) 6018 males (50.9%);<br>2) 5813 females (49.1%)	(M = 14.97, SD = 1.46)	Cross-sectional survey	Family suicide history	Perceived social support from family	1) Multidimensional Scale of Perceived Social Support (MSPSS) (Zimet et al., 1988);<br>2) "Have you ever seriously thought about suicide or killing yourself during the past 12 months?" - SI	1) Adolescents with family suicide history reported higher SI (χ^2=74.15, $P < 0.01$) as compared to those who did not report family suicide history.	1) Perceived social support from family was significantly and negatively correlated with SI (r = -0.108, $p < 0.001$);<br>2) in the mediation model, the direct and indirect effect of perceived social support from family on SI was significant.
Chae et al., (2020), South Korea	N = 2324	1) 1223 males (52.6%);<br>2) 1101 females (47.4%)	12-18 years	Cross-sectional survey	Parents' SI		1) "Have you considered ending your own life during the past year?" - asked both parents and adolescents	1) based on logistic regression, it was reported that the chances of adolescents' SI were twice as high when one of the parents also had SI (AOR = 2.01, 95% CI 1.32–3.05); 2)father with SI increased the chnaces of adolescent SI (AOR = 3.08, 95% CI [1.76, 5.40]).	
Peltzer & Pengpid, (2012), South Africa	N = 30284	1) 14688 males (48.5%);<br>2) 15596 females (51.5%)	13-15 years (M = 14.1, SD = 0.8)	Cross-sectional survey		1) Lack of parental support	Items were used from GSHS (CDC 2015):<br>1) 'During the past 30 days, how often did your parents or guardians check to see if your homework was done?' - Parental/guardian supervision;<br>2) 'During the past 30 days, how often did your parents or guardians understand your problems and worries?' - parental/guardian connectedness;<br>3) 'During the past 30 days, how often did your parents or guardians really know what you were doing with your free time?' - parental/guardian bonding;<br>4) 'During the past 12 months, did you ever seriously consider attempting suicide?' - SI		1) Using multivariate regression, it was reported that lack of parental support was significantly associated with SI (AOR = 0.47, 95% CI [0.36–0.62]).
Peng et al., (2024), China	N = 1809	1) 855 males (47.3%);<br>2) 954 females (52.7%)	12-18 years (M = 14.83, SD = 1.93)	Cross-sectional survey		Perceived social support from family	1) Perceived social support scale (PSSS) (Zimet et al., 1988);<br>2) Suicidal ideation is measured by the PANSI (Osman et al., 1998)		1) this study highlighted that family support had the strongest protective effect against adolescent SI (strength = 0.87, predictability = 0.53)
Kwok et al., (2009), China	N = 5557	1) 2951 males (53.1%);<br>2) 2606 females (46.9%)	11-18 years (M = 13.87, SD = 1.47)	Cross-sectional survey		Family functioning	1) Chinese Family Assessment Instrument (C-FAI) (Shel, 2000);<br>2) Suicidal Ideation Sub-Scale (C-SIS) of the Suicidal Risk Scale for Hong Kong students by Tse and Bagley (2002)		1) the family functioning (r = -0.46, $p < 0.0083$) and five subscales of family functioning: mutuality (r = -0.39, $p < 0.0083$), cpmmunication (r = -0.36, $p < 0.0083$), conflict and harmony (r = -0.44, $p < 0.0083$), parental concern (r = -0.39, $p < 0.0083$) and parental control (r = 0.39, $p < 0.0083$) were significantly and negatively associated with SI;<br>2) among all the subscales, parental concern (r = -0.17, $p < 0.001$) and parental control (r = -0.17, $p < 0.001$) were strongest predictors of SI;<br>3) family functioning as a mediator, was only found to weaken the reationship between social problem solving and SI among male adolescents (β = 0.47, $p < 0.05$) and not female adolescents.

Asante et al., (2021), Sierra Leone	N = 2798	1) 1258 males (45%);<br>2) 1484 females (53%);<br>3) 56 Unknown (2%)	17-18 years	Cross-sectional survey	1) Parental monitoring;<br>2) Parental intrusion of privacy	1) Parental understanding;<br>2) Parental supervision	1) “during the past 12 months, did you ever seriously consider attempting suicide?” - SI;<br>2) WHO-GSHS questionnaire consists of validated survey items examining the behavioural risks and protective factors across multiple areas of functioning amongst school-going adolescents.	1) based on bivariate analysis, among the two risk factors only parental monitoring was significantly and positively associated with adolescent SI (β = 0.491, AOR = 1.63, 95% CI [1.26, 2.12], p < 0.001).	1) based on bivariate analysis, only parental supervision was significantly and negatively associated with SI (β = -0.831, AOR = 0.44, 95% CI [0.34, 0.57], p < 0.001);<br>2) based on multivariable associations, only parental supervision had a protective effect against SI (OR: 0.37, 95% CI = 0.27–0.51; p < 0.001).
Bao et al., (2024), China	N = 3443	1) 1818 males (52.8%);<br>2) 1625 females (47.2%)	12-18 years (M = 14.88, SD = 1.59)	Cross-sectional survey		1) Family social support	1) perceived social support scale (PSSS) (Zimet et al., 1988);<br>2) PHQ-9 - SI (Kroenke et al., 2001)		1) using binary logistic regression, parental social support was reported to be protective against SI (OR = 0.888, 95 % CI: 0.859–0.918, p < 0.001);<br>2) family support as a mediator also mediated the relationship between anxiety and SI, with high family support reducing SI risk (b = –0.366, 95% CI: –0.474, -0.404, R2 = 0.321).
Yang et al., (2023), China	N = 3267	1) 1665 males (51%);<br>2) 1602 females (49%)		Cross-sectional survey		Family social support	1) Multidimensional Scale of Perceived Social Support (MSPSS) (Zimet et al., 1988);<br>2) Suicidal ideation was evaluated based on one item from the Chinese version of the Children’s Depression Inventory (CDI) (Kovacs, 1991; Wu et al., 2010).		1) in the logistic regression analysis, low perceived social support at baseline also significantly predicted SI at the follow-up of 6 months (OR =1.78; 95% CI: 1.10–2.87)
Walsh et al., (2012), Israel	N = 200	1) 96 males (48%);<br>2) 104 females (52%)	15-18 years	Cross-sectional survey		Parental support	1) Three items were used to measure parental support:<br>a) ‘To what extent can you turn to your parents when you are dealing with something difficult?’;<br>b) ‘To what extent do your parents give you support when you are distressed or dealing with a problem?’;<br>c) ‘To what extent do you feel your parents can help you when you are having a hard time?’;<br>2) SI was measured using 4 items based on questionnaires of Wilburn and Smith (2005) and Roberts, Roberts, and Chen (1997):<br>a) ‘I thought of committing suicide,’<br>b) ‘I thought of how I would commit suicide,’<br>c) ‘I thought of when I would commit suicide,’<br>d) ‘I thought that if I had the opportunity I would commit suicide.’		1) Parental support had a significant negative association with SI (r = -0.361, p < 0.01).
Pin˜a-Watson et al., (2014), USA	N = 345	1) 0 males (0%);<br>2) 345 females (100%)	13-18 years	Longitudinal		1) Mother connectedness;<br>2) Father connectedness;<br>3) Autonomy;<br>4) Parental academic interest;<br>5) Parental caring	1) Mother connectedness scale;<br>2) Father connectedness scale;<br>3) Autonomy granting scale;<br>4) Parental academic interest scale;<br>5) Parental caring scale;<br>6) ‘‘During the past 12 months, did you ever seriously think about committing suicide?’’ - SI		1) two logistic regression models were used to determine significant parental predictors of SI;<br>2) except for autonomy (r = 0.03, p > 0.05), parental caring (r = 0.31, p < 0.01), mother (r = 0.25, p < 0.01) and father connectedness (r = 0.22, p < 0.01), parental academic interest (r = 0.23, p < 0.01) had a significant negative correlation with SI;<br>3) among all the predictors related to parental factors in model 1, which only studied adolescents with both parents, parental academic interest was the only factor that significantly predicted SI (B = 0.39, p < 0.05);<br>4) the odds of SI increased by a unit of 0.68 with parental academic interest;<br>5) for model 2, where only adolescents with one parent were studied, academic interest (B = -0.34, p < 0.05) and parental caring (B = -0.18, p < 0.01) were the statistically significant predictors of SI;<br>6) the odds of participants reporting SI increased by an increase in the unit of parental academic interest and caring by 0.71 and 0.83, respectively.
Gouveia-Pereira et al., (2014), Portugal	N = 534	1) 261 males (48.9%);<br>2) 273 females (51.1%)	14-18 years (M = 16.18, SD = 1.14)	Cross-sectional survey		1) Family functioning;<br>2) Conflictual independence	1) Family Adaptability and Cohesion Evaluation Scale (FACES III) developed by Olson, Portner, and Lavee (1985);<br>2) Psychological Separation Inventory – Conflictual Independence Hoffman (1984) and adapted by Santos (2001) to a sample of Portuguese adolescents;<br>3) Suicidal Ideation scale, adapted by Ferreira and Castela (1999)		1) results from two-way ANOVA stated that adolescents from unbalanced families (M = 2.33, SD = 1.15) reported higher SI, as compared to those from balanced (M = 1.61, SD = 0.67) and midrange families (M = 1.80, SD = 0.92);<br>2) high conflictual independence from mother (M = 1.73, SD = 0.87) and father reported low SI.

Guan et al., (2024), China	N = 877	1) 419 males (47.8%);<br>2) 458 females (52.2%)	12-15 years (M = 13.80, SD = 0.97)	Cross-sectional survey	Negative parenting style		1) Parenting Styles Questionnaire developed by Arrindell et al. (1999) and revised by Jiang et al. (2010);<br>2) Self-rating Idea of Suicide Scale, compiled by Xia et al. (2012).	1) negative parental parenting style ($r = 0.27, p < 0.001$) and negative maternal parenting style ($r = 0.34, p < 0.001$) had significant positive association with SI;<br>2) with negative emotions as mediator, negative parental parenting style positively predicted SI both directly ($\beta = 0.27, p < 0.001$) and indirectly ($\beta = 0.13, p < 0.001$);<br>3) similarly, with negative emotions as mediator, negative maternal parenting style positively predicted SI both directly ($\beta = 0.32, p < 0.001$) and indirectly ($\beta = 0.19, p < 0.001$).	
Cho, (2020), South Korea	N = 458	1) 220 males (48%);<br>2) 238 females (52%)	11-13 years	Cross-sectional survey	Parental abuse	1) Parental supervision and guidance;<br>2) parents' participation in education	1) a four-item scale developed by Lee and Yang (2003) - parents' participation in education;<br>2) a four-item scale developed by Lee and Yang (2003) - parental supervision and guidance;<br>3) a four-item scale developed with reference to the Seoul Child Panel (2005) - parental abuse;<br>4) Six items from the Suicidal Ideation Questionnaire by Reynolds (1988) - Suicidal Ideation.		1) based on hierarchical logistic regression, the presence of a parental figure negatively predicted SI ($\beta = -3.392, p < 0.01$);<br>2) throughout all the steps of hierarcical logistic regression, presence of parental figure remained a significant predictor of SI in step 2 ($\beta = -3.243, p < 0.01$) and step 3 ($\beta = -2.567, p < 0.01$);<br>3) with being introduced in step 3, only parental supervision and guidance signifiantly predicted SI ($\beta = -0.177, p < 0.01$).
Li et al., (2025), China	N = 6474	1) 3039 males (46.9%);<br>2) 3435 females (53.1%)	12-19 years (M = 15.86, SD = 1.79)	Cross-sectional survey	Parental psychological control		1) Parental Psychological Control Scale-Youth Version (PCS-YSR) developed (Choi & Kim, 2018);<br>2) Self-Assessment of Suicidal Ideation Scale (SASI) was developed by Xia and Wang(2002).	1) parental psychological control had a significant positive association with SI ($r = 0.308, p < 0.001$);<br>2) in a chain mediation model, with basic psychological need and meaning in life as mediators, parental psychological control positively and significantly predicted SI ($\beta = 0.10, p < 0.001$).	
Potard et al., (2020), France	N = 455	1) 143 males (31.4%);<br>2) 312 females (68.6%)	12-18 years (M = 15.78, SD = 1.61)	Cross-sectional survey	separation anxiety	Parental attachment	1) Inventory of Parent and Peer Attachment (IPPA; Vignoli & Mallet, 2004);<br>2) Suicidal Ideation Questionnaire (SIQ-Fr; Potard, Kubiszewski, Gimenes, & Courtois, 2014);<br>3) Separation Anxiety Symptom Inventory (SASI; Brandibas, Sudres, Gaspard, & Jeunier, 2010)	1) separation anxiety was significantly and positively associated with SI for both boys and girls;<br>2) separation anxiety partially mediated the relation between poor maternal attachment and SI in both sexes;<br>3) separation anxiety partially mediated the relation between poor paternal attachment and SI among boys only.	1) poor parental attachment was significantly associated with higher SI for both boys and girls;<br>2) greater maternal attachment insecurity was associated with high SI risk in both boys and girls;<br>3) greater paternal attachment insecurity was associated with high SI risk in boys only
Guo et al., (2021), China	N = 8680	1) 4618 males (53.2%);<br>2) 4062 females (46.8%)	12 -19 years (M = 15.224, SD = 2.559)	Cross-sectional survey		Parental attachment	1) positive and negative suicide ideation (PANSI) inventory (Osman et al., 1998);<br>2) inventory of parent and peer attachment (IPPA) (Armsden and Greenberg, 1987) - Parental attachment		1) Parental attachment was significantly associated with SI (r PANSI-PSI*IPPA-Father = −0.439, r PANSI-PSI*IPPA Mother = −0.485, r PANSI-NSI*IPPA-Father = −0.322, r PANSI-NSI*IPPA-Mother = −0.352);<br>2) in SEM, parental attached had a significant and negative total effect on SI ($\beta = -0.137$, $Z = -27.00$, 95% CI [−0.147, −0.127], $p < 0.001$);<br>3) as for the direct effect, parental attachment had a significant negative direct effect on SI ($\beta = -0.107$, $Z = -21.40$, 95% CI [−0.117, −0.098], $p < 0.001$);<br>4) the indirect effect of parental attachemnt via trait anhedonia ($\beta = -0.019$, $Z = -19.00$, 95% CI [−0.022, −0.017], $p < 0.001$) and peer attachment ($\beta = -0.008$, $Z = -7.00$, 95% CI [−0.010, −0.007], $p < 0.001$) on SI was also significantly negative;<br>5) the direct and indirect effect suggests that healthy parental attachment is linked to lower risk of SI.
Wang & Qiao, (2022), China	N = 2407	1) 1198 males (49.77%);<br>2) 1209 females (50.23%)	11-16 years (M = 12.75, SD = 0.58)	Longitudinal	Parental Phubbing		1) Parental Phubbing Scale revised by Wang et al., (2020);<br>2) Positive and Negative Suicide Ideation Scale - SI.	1) parental phubbing had a positive significant association with SI at T1 and T2 ($r_{\square 2}$=0.067, p<0.05, $r_{\square 3}$=0.16 p<0.001, respectively);<br>2) the cross-lagged analysis reported T1 parental phubbing to be significantly and positively predicting T2 SI (β=0.083, p<0.001), but not from T2 parental phubbing to T3 SI (β=0.030,p=0.22);<br>3) self-esteem at T2 as a mediator positively and significantly predicted the effect of parental phubbing at T1 on SI at T3 ($\beta = 0.013$, (95%CI = [0.003, 0.028]);<br>4) the path between parental phubbing and SI was significant for adolescents who scored low on extraversion ($\beta = 0.16, p < 0.001$), but insignificant for those who scored high on extraversion ($\beta = 0.015, p = 0.67$), this indicated that adolescents with high perceived parental phubbing and low extraversion have high risk of SI.	

Wang et al., (2014), Taiwan	N = 5051	1) 2179 males (43.14%);<br>2)2872 females (56.86%)	1) M = 15.17, SD = 1.66 - No problematic cellular phone use (CPU);<br>2) M = 15.25, SD = 1.70 - Problematic cellular phone use (CPU)	Cross-sectional survey		1) Family functioning	1) Kiddie-Schedule for Affective Disorders and Schizophrenia (K-SADS-E) questionnaire - SI (1-4 item number);<br>2) Family APGAR Index (APGAR) - Taiwanese version to measure family functioning		1) with family functioning used as a moderator, the association between problematic CPU and SI was lower in the logistic regression model (OR = 0.92, p < 0.001, 95% CI [0.90, 0.94]);<br>2) this may be interpreted as adolescents with problematic CPU exhibits lower risk of SI due to higher levels of family functioning.
Bar-Zomer & Brunstein Klomek, (2018), Israel	N = 279	1) 115 males (41.22%);<br>2) 164 females (58.78%)	10-17 years (M = 13.5, SD = 1.9)	Cross-sectional survey	Sibling bullying	Parental attachment	1) the bullying questionnaire - adapted to measure sibling bullying (Sourander et al., 2010);<br>2) the Attachment Security Style Scale (Kerns et al., 1996);<br>3) Mood and Feelings Questionnaire (Angold, 1995) - four items for SI	1) a significant association was reported between sibling bullying and SI ($\chi2(1)$ = 10.436, p < 0.005);<br>2) based on multiple logistic regression, with secure attachment to mother in the model, involvement in sibling bullying did not significant association with SI (B = 0.68, Z = 1.80, OR = 1.98, ns);<br>3) similarly, with secure attachment to father in the model, involvement in sibling bullying had a significant association with SI (B = 0.84, Z = 2.14, OR = 2.31, p < 0.05).	1) based on multiple logistic regression, secure attachment style to mother and father had a significant negative association with SI (B = −0. 05, Z = −2.15, OR = 0.96, p < 0.05; B = −0.06, Z = −3.48, OR = 0.94, p < 0.001, respectively);<br>2) attachment to mother as a moderator did not significantly moderate the association between sibling bullying and SI (B = −0.01, Z = −0.13, OR = 1.88, ns);<br>3) atachment to father as a moderator significantly moderated the association between involvement in sibling bullying and SI (B = 0.07, Wald = 0.04, OR = 2.35, p < 0.05).
Chen et al., (2020), China	N = 610	1) 338 males (55.41%);<br>2) 272 females (44.59%)	M = 15.03, SD = 1.69	Cross-sectional survey		Parental marital satisfaction	1) Parental Marital Satisfaction Scale in the Olson Marriage Quality Questionnaire (Fowers & Olson, 1989);<br>2) Beck Scale for Suicide Ideation (BSI) (Li et al., 2011)		1) parental marital satisfaction had a significant negative association with SI (r = -0.113, p < 0.01);<br>2) with short breastfeeding duration as a moderator, parental marital satisfaction had a significant predictive power on SI (B = −0.117, SE = 0.034, t = −3.425, p < .001), but the predictive power was insignificant with long breastfeeding duration (B = −0.021, SE = 0.034, t = −0.619, p = .54).
Wang et al., (2023), China	N = 857	1) 392 males (45.74%);<br>2) 465 females (54.26%)	11-18 years (M = 14.7, SD = 1.68)	Cross-sectional survey		Family incivility	1) Family Incivility Scale (Lim & Tai, 2014);<br>2) Suicidal Ideation Scale (Wang et al., 2011)	1) family incivility had a significant positive association with SI (r = 0.41, p < 0.01);<br>2) with hopelessness as a mediator in the model, the direct (γ = 0.14, p < .001) and indirect effect (b = 0.307, SE = 0.038, 95% CI = [0.31, 0.45], p < . 001) of family incivility via hopelessness on SI were significant;<br>3) family incivility had an indirect significant effect on SI through hopelessless, with self-esteem as a moderator (b = 0.22, SE = 0.03, 95% CI = [0.17, 0.27] - low self-esteem; b = 0.07, SE = 0.02, 95% CI = [0.03, 0.12] - high self-esteem);<br>4) family incivility indirectly predicted SI strongly for girls (b = 0.39, SE = 0.044, 95% CI = [0.31, 0.48]), but this effect was weak for boys (b = 0.22, SE = 0.039, 95% CI = [0.15, 0.30]);<br>5) family incivility indirectly predicted SI via hopelessness for early (b = 0.32, SE = 0.044, 95% CI = [0.24, 0.41]) and middle adolescents (b = 0.29, SE = 0.038, 95% CI = [0.22, 0.36]).	
MacPherson et al., (2021), USA	N = 111	1) 64 males (57.66%);<br>2) 47 females (42.34%)	12-18 years (M = 15.73, SD = 1.19)	Randomized Controlled Trial		1) Parental monitoring;<br>2) Family functioning;<br>3) Parental processes	1) Parental Monitoring subscale of the Parental Monitoring Questionnaire (PMQ) (Stattin and Kerr, 2000);<br>2) Family Assessment Device (FAD) (Epstein et al., 1983) - family functioning;<br>3) Family Assessment Task (FAsTask) (Dishion et al., 2003) - parental processes	1) observed parental monitoring significantly predicted a decrease in SI over 12 12-month period (F (265.79) = 5.94, p = .015, d = 0.46);<br>2) other factors like parental listening, limit setting, self-reported monitoring, and family functioning did not predict SI over time.	
Kim et al., (2021), South Korea	N = 1150		13-14 years	Cross-sectional survey	Family violence		1) Conflict Tactics Scale (CTS) by Straus, 1990;<br>2) Scale of Suicidal Ideation (SSI) was developed by Beck et al., 1974, and Park and Shin et al., 1990.	1) exposure to family violence had a significant positive association with SI (r = 0.311, p < 0.01);<br>2) based on SEM, exposure to family violence had a significant effect on SI (direct effect - SE = 0.11, p = 0.005; indirect effect - SE = 0.18, p = 0.002; total effect - SE = 0.29, p = 0.002);<br>3) exposure to family violence also had a significant indirect effect (SE = 0.18, p = 0.002) via anger and depression.	

Kwok & Shek, (2009), China	N = 5557	1) 2951 males (53.10%);<br>2) 2606 females (46.90%)	11-18 years (M = 13.87, SD =1.47)	Cross-sectional survey		1) Parent-adolescent communication;	1) Father-Adolescent Communication Scale (FACS) and Mother-Adolescent Communication Scale (MACS) developed by Shek et al. (2006);<br>2) Suicidal Ideation Sub Scale (C-SIS) of the Suicidal Risk Scale for Hong Kong students developed by Tse (2002).		1) father-adolescent communication ($r = -0.36$, $p < 0.006$), mother-adolescent communication ($r = -0.42$, $p < 0.006$) had significant negative associations with male and female SI;<br>2) association between father-adolescent communication and SI was stronger for females ($r = 0.40$, $p < 0.006$) than males ($r = 0.33$, $p < 0.006$);<br>3) standard regression analysis reported mother-adolescent ($\beta = -0.34$, $p < 0.001$) and father-adolescent communication ($\beta = -0.23$, $p < 0.001$) predicting SI, with a variance of 23% in the sample;<br>4) mother-son communication had a stronger link with SI for males ($tH = 4.78$, $p < 0.001$) and maother-daughter communication for females ($tH = 1.99$, $p < 0.05$) based on regression analysis;<br>5) hopelessness moderated the association between father-adolescent cmmunication and mother-adolescent communication only for males ($\beta = -0.24$, $p < 0.05$; $\beta = -0.35$, $p < 0.01$, respectively).
Wu & Yaccob, (2016), Malaysia	N = 684	1) 305 males (44.6%);<br>2) 379 females (55.4%)	14-17 years (M = 15.3, SD = 0.810)	Cross-sectional survey		Parental closeness	1) Network of Relationships– Relationship Quality Version (NRI-RQV; Buhrmester & Furman, 2008);<br>2) Positive and Negative Suicide Ideation Inventory (PANSI; Osman, Gutierrez, Kopper, Barrios, & Chiros, 1998)		1) mother closeness ($r = -0.133$, $p < 0.01$) and father ($r = -0.093$, $p < 0.05$) closeness had a significant negative association with SI, indicating lower SI risk among those with better relationships with their parents;<br>2) in the mediation model, mother closeness ($B = .0448$, $t(682) = 3.4965$, $p < .001$) and father closeness ($B = .0287$, $t(682) = 2.4275$, $p < .05$) had a significant direct effect on lower SI;<br>3) the indirect effect of mother closeness and father closeness on SI was significant both by sobel effect ($Z = 2.038$, $p < .05$; $Z = 2.361$, $p < .05$, respectively) and bootstrap method ($B = -0.0093$, $SE = 0.0047$, 95% CI = 0.0006 to 0.0181; $B = -0.0082$, $SE = 0.0037$, 95% CI = 0.0163 to 0.0017) indicating partial mediation.
Nunes & Mota, (2016), Portugal	N = 604	1) 274 males (45.4 %);<br>2) 330 females (54.6 %)	15-18 years (M = 15.99, SD = 0.97)	Cross-sectional survey		1) Parenting styles;<br>2) Parental attachment	1) Parenting Styles & Dimensions Questionnaire: Short Version (PSDQ) translated into Portuguese by Nunes and Mota (2013);<br>2) Father/Mother Attachment Questionnaire (FMAQ) (Matos and Costa, 2001);<br>3) Suicidal Ideation Questionnaire (SIQ) adapted by Ferreira and Castela (1999)		1) the direction of association between parental attachment and SI varied as per the different dimensions of the variables, for instance, the association between one of the dimensions of parental attachment (quality of emotional bond) and SI, the association was significantly negative for both father and mother ($r = -0.25$, $r = -0.31$), and the association between another dimension (exploration of individuality and exploraion) and SI was significantly positive for both father and mother ($r = 0.26$, $r = 0.28$);<br>2) similar results were noted for parenting styles, significant negative association was found between authoritative style and SI for both father and mother ($r = -0.09$, $r = -0.27$), and significant positive association with SI was found between authoritarian and permissive styles for both father and mother ($r = 0.11$, $r = 0.28$);<br>3) authoritative parenting was associated with more secure attachment ($\beta = -0.28$, 95% CI [−0.34, −0.22]), which in turn predicted lower suicidal ideation ($\beta = -0.15$, 95% CI [−0.21, −0.09]);<br>4) indirect effect of parenting style on SI through attachment was significant (indirect $\beta = 0.04$, $SE = 0.01$, 95% CI [0.02, 0.06]);<br>5) direct path remained significant from parenting style to SI ($\beta = -0.08$, 95% CI [−0.13, −0.03]).

Kelly et al., (2025), USA	N = 2118	1) 1046 males (49.38%);<br>2) 1027 females (48.49%);<br>3) 5 trans male (0.24%);<br>4) 2 trans female (0.09%);<br>5) 16 non-binary (0.76%);<br>6) 6 gender non-conforming (0.28%);<br>7) 16 missing (0.76%)	12-18 years (M = 15.97, SD = 1.57)	Cross-sectional survey		1) Perceived social support from family;<br>2) perceived family support	1) The Social Support for Adolescents Scale by Cauce et al. (1982);<br>2) Communities that Care Community Survey - perceived family support;<br>3) single item from the Youth Risk Behavior Surveillance Survey (YRBSS) - SI		1) perceived family support had a significant negative correlation with SI (r = -0.14, p < 0.01);<br>2) based on logistic regression analysis in model 1, perceived family support had an association with 13% lower odds of SI risk (eB/OR = 0.87, 95% CI = 0.82, 0.90)and in model 3 and 4, perceived family support reported lower odds of SI (eB/OR = 0.86, 95% CI =0.82, 0.90; eB/OR = 0.87, 95% CI = 0.82, 0.90, respectively).
Nieto-Casado et al., (2022), Spain	N = 1405	1) 659 males (46.9%);<br>2) 746 females (53.1%)	12-17 years (M = 14.79, SD = 1.74)	Cross-sectional survey		parental competences (i.e., parental warmth, family communication, and autonomy support)	1) Acceptance-Rejection subscale of the Child Report on Parenting Behavior Inventory (CRPBI; Barber et al., 2005; Schaefer, 1965) - Parental warmth;<br>2) Family Communication Scale IV (FCS; Olson et al., 2006; validated by Sanz et al., 2002) - family communication;<br>3) autonomy support subscale from the instrument Perceptions of Parents Scale (POPS; Grolnick et al., 1991; Soenens et al., 2007) - autonomy support;<br>4) negative subscale of the Positive and Negative Suicide Ideation Inventory (PANSI; Osman et al., 1998; validated by Villalobos-Galvis, 2009)		1) based on SEM, parental competencies had a significant direct association with reduced SI (β = -0.14, p < 0.001);<br>2) with mindfulness as a mediator, parental competencies did not predict SI (β < 0.01, p = 0.91);<br>3) the indirect effect of parental competencies on SI through anxious-depressive symptoms (β = -0.09, p < 0.01) and through serial mediation of mindfulness and anxious-depressive symptoms (β = -0.10, p < 0.01) were significant.
Nakano, (2021), Japan	N = 379	1) 195 males (51.45%);<br>2) 184 females (48.55%)	13-15 years (M = , SD =)	Cross-sectional survey		Family support	1) "Do you feel that you are supported by your school staff/family members?" - family support;<br>2) "Did you think that you feel like committing suicide?" - SI		1) support from family members was a protective factor against SI;<br>2) based on Fisher's test it was noted that SI risk was higher by 77.8% for those with low family support, as compared to those with high family support, where the SI risk is about 32% (p = 0.025);
Modeste-James et al., (2023), USA	N = 3840	1) 1790 males (46.6%);<br>2) 2050 females (53.4%)	14-18 years (M = , SD =)	Cross-sectional survey	Parental invasion of privacy	Parental understanding	1) During the past 30 days, how often did your parents or guardians go through your things without your approval? - parental invasion of privacy;<br>2) During the past 30 days, how often did your parents or guardians understand your problems and worries? - parental understanding;<br>3) "During the past 12 months, did you ever seriously consider attempting suicide?" - SI	1) based on bivariate analysis and logistic regression analysis parental invasion of privacy was not a significant predictor of SI;	1) parental understanding had a significant negative association with SI;<br>2) based on logistic regression, adolescents with parental understanding reported low SI risk (OR = 0.53, 95% CI = [0.42, 0.68], p < 0.001);<br>3) protective role of parental understanding against SI was only significant for females (OR = 0.42, p < .001, 95% CI = [0.35, .57]).
Peláez-Fernández et al., (2024), Spain	N = 2722	1) 1255 males (46.1%);<br>2) 1437 females (52.8%);<br>3) 8 non-binary (0.3%);<br>4) 22 non-reported (0.8%)	12-18 years (M = 14.11, SD = 1.42)	Cross-sectional survey		1) Family climate	1) Family Environment Scale (FES; Fernández-Ballesteros & Sierra, 1989) - Spanish version (family climate);<br>2) Suicidal Ideation Inventory (FSII; Chang & Chang, 2016; Sánchez-Álvarez et al., 2020) - Spanish version (SI)		1) among the three dimensions of family climate, cohesion (r = -0.29, p < 0.01) and expressiveness (r = -0.21, p < 0.01) had a significant negative association with SI, whereas conflict (r = 0.22, p < 0.01) was positively and significantly associated with SI;<br>2) based on regression analysis, all three dimensions - cohesion (β = 0.16, t = 6.60, p < 0.001), expresiveness (β = 0.12, t = 4.92, p < 0.001) and conflict (β = -0.18, t = -10.82, p < 0.001) were reported as significant predictors of SI;<br>3) moderation analysis showed that all the dimesions of family climate significantly moderated the impact of emotional intelligence (EI) on SI;<br>4) the impact of EI on SI became negative for those who scored low in cohesion (b = − 0.27, p < 0.001), and expressiveness (b = − 0.27, p < 0.001) as compared to those who scored high in cohesion (b = − 0.14, p < 0.001) and expressiveness (b = − 0.15, p < 0.001);<br>5) on the contrary the negative association between EL and SI was weaker for those who scored low in conflict (b = − 0.16, p < 0.001) than those who scored high in conflict (b = − 0.26, p < 0.001)
Ibrahim et al., (2019), Malaysia	N = 176	1) 105 males (60%);<br>2) 71 females (40%)	12-19 years (M = , SD =)	Cross-sectional survey		Family support	1) Multidimensional scale of perceived social support (MSPSS) by Zimet, Powell, Farley, Werkman & Berkoff in 1990;<br>2) Suicide Ideation Scale (SIS) (Rudd, 1989)		1) family support had a significant negative correlation with SI (r = -0.21, p < 0.01);<br>2) regression analysis reported family support to be a significant predictor of SI (b = -0.15, t = -2.07, p = 0.040), indicating it to be a protective factor against SI.

Kim et al., (2021), USA	N = 14272	1) 7288 males (51.1%); 2) 6984 females (48.9%)	12-17 years (M = 14.52, SD = 1.67)	Cross-sectional survey		Parental monitoring	1) seven items from NSDUH data were combined (SAMHSA, 2017) - Parental monitoring; 2) Three items from NSDUH data (SAMHSA, 2017) - SI: a) "During the period of time when problems were the worst, did you often think about death?" b) "Did you think that it would be better if you were dead?" c) "Did you think about killing yourself?"		1) parental monitoring had a direct significant negative association with SI (b = –.31, p < .001); 2) parental monitering as a moderator at all three levels, significantly interacted with depression to predict SI (b = 0.85, p < .001 - low; b = 0.74, p <.001 - mean; b = 0.62, p < .001 - high); 3) the mediating effect of depression on SI through social support was wearker for those experiencing parental monitoring at all levels (b = –.06, 95% CI [–.07,–.06] - low; b = –.06, 95% CI [–.06,–.05] - mean; b = -0.05, 95% CI [–.05,–.04])
Wang et al., (2023), China	N = 4144	1) 2172 males (52.41%); 2) 1972 females (47.59%)	(M = 16.41, SD = 0.77)	Cross-sectional study	Mother phubbing	Perceived acceptance from mother	1) Generic Scale of Being Phubbed (Chotpitayasunondh & Douglas, 2018) - Mother phubbing; 2) Perceived Acceptance Scale pertaining to mothers developed by Brock et al. (1998), adapted in Chinese (Qu et al., 2022); 3) "I thought about killing myself during the past 6 months." - SI adapted from Achenbach's (1991) Youth Self-Report Questionnaire	1) Mother phubbing had a significant positive association with SI (r = 0.21, p < 0.001); 2) In the mediation model, the direct effect of mother phubbing was significantly associated with SI (β = 0.20, p < 0.001); 3) with perceived acceptance of mother as mediator, mother phubbing had a significant effect on SI (β = 0.05, p < 0.001)	1) Perceived acceptance from the mother had a significant negative association with SI (r = − 0.29, p < 0.001); 2) in the mediation model, perceived acceptance of the mother had a significant effect on SI (β = − 0.10, p < 0.001); 3) with gender as moderator, the interaction effect of acceptance and gender had a significant moderating effect on SI (β = 0.04, p < 0.01); 4) this moderating effect was stronger in boys (β = − 0.14, p < 0.001) than among girls (β = − 0.06, p < 0.01).
Galindo-Dominguez & Losada Iglesias, (2023), Spain	N = 898	1) 448 males (49.89%); 2) 450 females (50.11%)	(M = 13.55, SD = 1.26)	Cross-sectional study		Family social support	1) Scale of Perceived Social Support (Arechabala & Miranda, 2002); 2) Suicide Risk Inventory for Adolescents- A Version (Hern´andez & Lucio, 2006)		1) family support significantly and negatively predicted SI (β = .485; p < .001), i.e., high family support led to a significant reduction in SI; 2) sex as a moderator interacted with family support and significantly moderated the relationship between family support and SI (β = .571; p < .001); 3) impact of family support in reducing SI was stronger in females (β = .582; p < .001) than in males (β = .320; p < .001).
Eslava et al., (2023), Spain	N = 229	1) 121 males (52.8%); 2) 108 females (47.2%)	14-17 years (M = 15.76, SD = 1.24)	Cross-sectional study	Family conflict		1) Strategic Family Evaluation (EFE) (Morell-Gomis et al., 2011); 2) Paykel Suicide Scale (PSS) (Fonseca-Pedrero et al., 2017; Paykel et al., 1974)	1) Family conflict had a significant positive association with SI (r = 0.21, p < 0.01); 2) based on mediation analysis, both the direct (b = 0.03, p = 0.042) and indirect effects (b = 0.08) of family conflict through assertive interpersonal schema significantly predicted SI.	
Quarshie et al., (2023), Namibia	N = 3152	1) 1380 males (44.3%); 2) 1772 females (55.7%)	(M = 15.1, SD = 1.4)	Cross-sectional study	1) Parental intrusion of privacy; 2) Food insecurity	1) Parental supervision; 2) Parental understanding; 3) Parental monitoring	1) WHO-GSHS - family level variables; 2) "during the past 12 months, did you ever seriously consider attempting suicide?" - SI	1) based on bivariate associations, both the risk factors, parental intrusion of privacy (χ2 = 16, p = 0.002) and food insecurity (χ2 = 13.9, p = 0.001), were significantly associated with SI; 2) based on logistic regression analysis, only parental intrusion of privacy (β = 0.29, AOR [95% CI] = 1.34 [1.04, 1.73], p = 0.024) significantly predicted SI;	1) based on bivariate associations, logictic regression and multinomial logistic regression analysis, none of the protective factors were significantly related to SI.
Bao et al., (2020), China	N = 2327	1) 1115 males (47.92%); 2) 1212 females (52.08%)	(M = 14.86, SD = 1.83)	Cross-sectional study		Family togetherness	1) Five statements were used to assess family togetherness: a) "My parents and I support each other during difficult times"; b) "We give each other plenty of time and attention"; c) "We talk openly and listen to each other"; d) "We feel loved and cared for by each other"; e) "We do things together and work together."		1) Family togetherness had a significant negative association with SI (r=−0.192, p < 0.001); 2) family togetherness as a moderator interacted with psychological pain, moderated the impact of psychological pain on SI (β = −0.216, p < 0.001); 3) on the other hand, family togetherness did not significantly moderate the relationship between being bullied and SI (β = 0.147, p > 0.05); 4) the moderated effect of family togetherness, in the indirect relationship of being bullied on SI through psychological pain was strongest for high family togetherness (b = 0.257, 95% CI: [0.080, 0.396]), significant but weak for average family togetherness (b = 0.149, 95%CI: [0.024, 0.242]) and non-significant for low famiily togetherness (b = 0.042, 95% CI: [−0.060, 0.133]).
Arango et al., (2018), USA	N = 142	1) 36 males (25.4%); 2) 106 females (74.6%)	12-15 years (M = 13.41, SD = 1.12)	Longitudinal survey		1) Family connectedness	1) Parent–Family Connectedness Scale (Resnick et al., 1997); 2) Suicidal Ideation Questionnaire–Junior (SIQ-JR; Reynolds, 1987)		1) the study was conducted longitudinally, once at baseline and a follow-up study after 6 months, at baseline and at 6 months follow-up family connectedness had a significant negative association with SI (r = -0.36, p < 0.05; r = -0.38, p < 0.05).

Ahouanse et al., (2022), China	N = 4732	1) 2359 males (49.85%);<br>2) 2373 females (50.15%)	10-18 years (M = 13.46, SD = 1.95)	Cross-sectional survey		Parental social support	1) Chinese version of Child and Adolescent Social Support Scale (CASSS) (Malecki & Demary, 2002) - Perceived social support;<br>2) Chinese version of the Beck Scale for Suicide Ideation (BSSI) - for 1 week and lifetime SI;<br>3) "how many times in the past year have you seriously considered ending your life?" - 1 year SI		1) Parental support was negatively associated with 1-week (AOR = 0.67, 95% CI = [0.55, 0.83]), 1-year (AOR = 0.64, 95% CI = [0.53, 0.77]) and lifetime SI (AOR = 0.63, 95% CI = [0.52, 0.77]);<br>2) parental support as a mediator in path analysis, significantly mediated the association between emotional neglect and amotional abuse in children for all types of SI, i.e., for 1-week SI by 5.31% and 29.23%, 1-year SI by 4.80% and 24.50% and lifetime SI by 7.04% and 44.42%, respectively.
Stone et al., (2015), USA	N = 2290	1) 1143 males (52.71%);<br>2) 1147 females (47.29%)	(M = 16.27, SD = 0.07)	Cross-sectional survey		Family connectedness	1) "Do you agree or disagree that your family loves you and gives you help and support when you need it?" - Family connectedness;<br>2) "During the past 12 months, did you ever seriously consider attempting suicide?" - SI		1) youth with high family connectedness reported low risk of SI as compared to those with low family connectedness;<br>2) in a model with all the variables, family connectedness was inversely associated with SI (OR = 0.32, 95% CI = [0.23, 0.44], p < 0.001).
Sarfo et al., (2023), Saint Lucia	N = 1864	1) 865 males (46.41%);<br>2) 999 females (53.59%)	13-17 years (M = , SD =)	Cross-sectional survey	Parental use of tobacco	Understanding parents	1) Do you have parents or guardians who use any form of tobacco? - Parental use of tobacco;<br>2) In the past 12 months, did you ever seriously consider attempting suicide? - SI	1) Students with parents/guardians using tobacco experienced an increased risk of SI (5.6%)	1) parental understanding was associated with lower risk of SI (p < 0.001);<br>2) based on logistic regression, adolescents with understanding parents were less likely to report SI (AOR = 0.627, 95% CI = [0.482, 0.816], p < 0.001)
Rubio et al., (2020), Chile	N = 775	1) 419 males (54.10%);<br>2) 356 females (45.90%)	(M = 15.48, SD = 0.96)	Cross-sectional survey		Family support	1) scale of perceived social support developed by Zimet et al., (1988) and validated by Mosqueda Díaz et al., (2015);<br>2) "I think about death or suicide" - SI		1) perceived family support had a significant negative association with SI (r = -0.35, p < 0.01);<br>2) perceived family support as a moderator, interacted with depression and significantly moderated the association between depression and SI (b = -0.03, 95% CI = [0.05, 0.02], p < 0.001).
Kaminski et al., (2009), USA	N = 4131	1) 2004 males (48.5%);<br>2) 2127 females (51.5%)	12-18 years (M = 15.08, SD = 1.92)	Cross-sectional survey		1) Family structure;<br>2) Family connectedness	1) Vaux Social Support Record (Vaux 1988) - Family connectedness;<br>2) a list of adult relatives (e.g., mother, stepfather, grandmother, uncle, etc.) was used to ask about family structure;<br>3) "During the past 12 months, did you ever seriously consider attempting suicide?" - taken from Youth Risk Behavior Survey (CDC 2003) - SI		1) based on logistic regression analysis, family connectedness was one of the strongest and significant protective factors against SI (OR = 0.54, P < 0.001, 95% CI [0.48, 0.61]);<br>2) based on multvariate models, family connectedness significantly and negatively predicted SI (AOR = 0.46, P < 0.001, 95% CI [0.40, 0.51]);<br>3) in the full model logistic regression, family connectedness had a strong negative association with SI (AOR = 0.60, P < 0.001, 95% CI [0.49, 0.73])
Barzilay et al., (2017), European countries (Austria, Estonia, France, Germany, Hungary, Ireland, Italy, Romania, Slovenia, and Spain)	N = 11110	1) males ();<br>2) females ()	15 years (M = 14.9, SD = 0.89)	Cross-sectional survey		Parental support	1) "during the past 2 weeks, have you reached the point where you seriously considered taking your life, or perhaps made plans how you would go about doing it?" - from Paykel Hierarchical Suicidal Ladder (Paykel, 1974) - SI;<br>2) Global School-Based Student Health Survey (WHO, 2010) - Parental support		1) based on logistic regression analysis, parental support as a moderated interacted only with verbal victimization and moderated effect on SI (OR = 1.11, 95% CI [1.01, 1.23]), i.e., if verbally victimized adolescents had high parental support, they reported low SI (OR = 0.95, 95% CI [0.82, 1.08], and if verbally victimized adolescents had low parental support SI increased by 63% (OR = 1.63, 95% CI [1.50, 1.76]);<br>2) additionally, risk of SI increased among whose verbally victimized adolescents who were depressed and had low parental support.
Ai et al., (2017), China	N = 2758	1) 1269 males (46%);<br>2) 1489 females (54%)	(M = 13.53, SD = 1.06)	Cross-sectional survey	Interparental conflict		1) Children's Perception of Marital Conflict Scale (Chi and Xin 2003);<br>2) "I thought about killing myself" from Youth Self-Report (Achenbach 1991) - SI	1) Interparental conflict had a significant positive association with SI (r = 0.18, p < 0.001);<br>2) using logistic regression, interparental conflict significantly predicted SI (B = 0.90, SE = 0.10, OR = 2.44, 95% CI [2.01, 3.00], p < 0.001);<br>3) with peer victimization as mediator in the multiple regression model, it partially mediated the direct association between interparental conflict and SI (B = 0.66, SE = 0.08, OR = 1.94, 95% CI [1.67,2.26], p < 0.001)	
Caballero-Domınguez & Campo-Arias, (2022), Colombia	N = 1462	1) 580 males (39.7%);<br>2) 882 females (60.3%)	13-17 years (M = 14.4, SD = 0.8)	Cross-sectional survey	Family dysfunction		1) YOUTH for middle- school students is a tool of the Center for Disease Control of the United States (Brener et al., 2013) - SI;<br>2) APGAR- adapted in Colombian adolescents (Arias & Herrera, 1994) - family dysfunction	1) Family dysfunction significantly and positively predicted SI based on crude association (OR = 6.74, 95% CI [4.32, 10.09]) and adjusted association (AOR = 4.54, 95% CI [2.35–8.77]) in logistic regression,	

Goodwill, (2020), USA	N = 2952	1) 720 males (24.4%);<br>2) 2232 females (75.6%)	12-17 years (M = , SD =)	Cross-sectional survey	Parental conflict	Verbal affirmation from parents	1) "During the past 12 months, how many times have you argued or had a fight with at least one of your parents?" - family conflict;<br>2) "During the past 12 months, how often did your parents let you know when you'd done a good job?" and "During the past 12 months, how often did your parents tell you they were proud of you for something you had done?" - verbal affirmation;<br>3) "Did you think about killing yourself?" - SI	1) fighthing with parents significantly predicted SI in both boys (OR = 1.18, 95% CI [1.08, 1.28]) and girls (OR = 1.17, 95% CI [1.02, 1.35]).	1) based on logistic regression, girls were reported to have 19% less chance to SI (OR = 0.81, 95% CI [0.71, 0.92]), but boys had no direct association with SI.
Jung & Cho, (2020), South Korea	N = 6316	1) 3162 males (50.1%);<br>2) 3154 females (49.9%)	13-18 years (M = 15.52, SD = 1.69)	Cross-sectional survey		Parental support	1) parental support scale developed by the NYPI (Mo et al. 2013) - Parental support;<br>2) "Have you ever thought abou killing yourself in the last year?" based on a study by Sohn (2014) - SI		1) low parental support in both boys and girls in terms of emotional (χ^2 = 112.57, p < 0.001), informational (χ^2 = 53.15, p < 0.001) and economic support (χ^2 = 31.74, p < 0.001) was associated with high rates of SI;<br>2) based on logistic regression, low emotional parental support significantly predicted SI in both males (OR = 0.46, p < 0.001) and females (OR = 0.40, p < 0.05), and only low parental informational support significantly predicted SI in females (OR = 1.87, p < 0.01).
Liu et al., (2024), China	N = 1913	1) 855 males (44.7%);<br>2) 1058 females (55.3%)	11-19 years (M = 14.86, SD = 1.61)	Cross-sectional survey		Family SES	1) parents' education, parents' occupation, and household income were asked to measure family SES; 2) Beck Scale for Suicide Ideation-Chinese Version (BSI-CV; Li et al., 2010) - integrating Suicide Ideation–current (SSI–C; Beck et al., 1979) and the Scale for Suicide Ideation–worst (SSI–W; Beck al., 1999)		1) family SES did not have a significant association with SI for both SI - current (r = -0.03, p > 0.01), SI - worst (r = -0.03, p > 0.01);<br>2) based on process analysis, family SES did not significantly predict SI, both SI - cuurent (B =-0.01, p >0.05, 95 % CI [-0.02, 0.01]), SI - worst (B =-0.01, p >0.05, 95 % CI [-0.01, 0.01]).
King et al., (2018), USA	N = 17399	1) 8613 males (50.5%);<br>2) 8786 females (49.5%)	12-17 years (M = , SD =)	Cross-sectional survey		Parental involvement	1) Participants rated their parents' frequency of involvement in seven behaviors via a four-point scale -<br>(a) checked whether homework is done;<br>(b) helped with their homework;<br>(c) made youth do chores around the house;<br>(d) limited the amount of time spent watching television;<br>(e) limited the amount of time spent with friends on school nights;<br>(f) told them they did a good job;<br>(g) and told them they were proud of them;<br>2) When problems were the worst did you think about killing yourself?		1) adolescents who reported high parental involvement in different domains such as - check if homework is done (OR = 2.795, 95% CI [2.477, 3.155], p < 0.001), help with homework (OR = 3.057, 95% CI [2.713, 3.444], p < 0.001), make youth do chores around the house (OR = 1.245, 95% CI [1.062, 1.461], p < 0.01), limit amount of TV (OR = 1.683, 95% CI [1.488, 1.904], p < 0.001), limit time out on a school night (OR = 1.263, 95% CI [1.120, 1.424], p < 0.001), tell youth they did a good job (OR = 3.496, 95% CI [3.095, 3.948], p < 0.001), tell youth they are proud of you (OR = 3.684, 95% CI [3.263, 4.159], p < 0.001) also reported low SI.
Sun & Ban, (2022), China	N = 2042	1) 933 males (45.7%);<br>2) 1109 females (54.3%)	13-16 years (M = 13.91, SD = 0.79)	Cross-sectional survey	Parental psychological control (PCC)		1) Parental psychological control with guilt induction, love withdrawal, and authority assertion subscales (Wang et al., 2007) - Parental psychological control;<br>2) SI of Chinese adolescents, including the subscales of despair, optimism, sleep, and lie (Xia et al., 2002) - SI	1) PCC had a significant positive association with SI (r = 0.39, p < 0.001);<br>2) based on mediation analysis with PCC as a predictor, it did not significantly predict SI (β = 0.02, p = 0.40, t = 0.85, 95% CI [-0.02, 0.05]);	0.02
Mulenga et al., (2017), Morocco	N = 2924	1) 1495 males (52.3%);<br>2) 1361 females (47.7%)	14-16 years (M = , SD =)	Cross-sectional survey		Parental understanding	1) Global School-based Health Survey (GSHS)		1) Based on logistic regression analysis, parental understanding reduced SI by 15% (AOR = 0.85, 95% CI [0.84, 0.85]).
Siziya et al., (2017), Samoa	N = 2418	1) 970 males (48.4%);<br>2) 1376 females (51.6%)	14-16 years (M = , SD =)	Cross-sectional survey		Parental understanding	1) Global School-based Health Survey (GSHS)		1) Based on logistic regression analysis, students with parental understanding were 19% (AOR = 0.81, 95% CI [0.74, 0.89]) less likely to report SI.
Aliy et al., (2023), Ethiopia	N = 1144	1) 431 males (37.7%);<br>2) 713 females (62.3%)	15-19 years (M = 16.9, SD = 1.96)	Cross-sectional survey	1) family history of suicide;<br>2) family history of suicide attempt	1) Familt environment;<br>2) parental understanding;		1) adolescents with family history of suicide attempts had higher risk of SI (AOR: 2.7; 95% CI: [1.7, 4.2]).	1) Based on logistic regression analysis, adolescents and school with non-suppportive family environment had higher risk of SI (AOR: 1.7; 95% CI: [1.1, 1.6], AOR: 1.7; 95% CI: [1.1, 2.6], respectively);<br>2) parental understanding reduced the risk of SI (AOR: 0.6; 95% CI: [0.4, 0.9])
Han et al., (2023), South Korea	N = 6512 families	1) 3471 males (53.2%);<br>2) 3041 females (46.8%)	12-18 years (M = 15.13, SD = 0.28)	Cross-sectional survey	1) parental depressive mood;<br>2) parental SI		1) "Have you seriously considered suicide in the past 12 months?" - Parental and adolescent SI;<br>2) "Have you experienced a continuous period of at least two weeks when you felt extremely sad or hopeless, to the point where it significantly affected your daily life in the past 12 months?" - Parental depressive mood	1) adolescents reporting parents with mental health issues had higher odds of SI, such that mother reporting diagnosed depression (OR = 2.109, 95% CI = [1.023, 4.350]), depressed mood (OR = 2.155, 95% CI = [1.224, 3.793]) and SI (OR = 2.532, 95% CI = [1.322, 4.851]) was associated with adolescent SI and only fathers' reporting SI (OR = 4.295, 95% CI = [1.747, 10.599]) was associated with adolescent SI.	

Wu et al., (2023), China	N = 417	1) 240 males (57.55%);<br>2) 177 females (42.45%)	12-18 years (M = 14.84, SD = 1.08)	Longitudinal survey		Family cohesion	1) Family Adaptability Cohesion Scale, Second Edition, Chinese version (FACES II-CV) (Olson et al., 1982; Fei et al., 1991);<br>2) Self-rating Idea of Suicide Scale (SIOSS; Xia et al., 2002)		1) family cohesion as a moderator significantly interacted with peer victimization and impacted SI (β = 0.116, SE = 0.045, $p < 0.05$, 95 % CI = [0.204, 0.025]);<br>2) the interation effect between family cohesion and psychological suzhi on SI is not significant.
Cong et al., (2020), Malaysia	N = 852	1) 431 males (50.6%);<br>2) 421 females (49.4%)	13-17 years (M = 14.8, SD = 1.225)	Cross-sectional survey		Family functioning	1) Family Adaptability and Cohesion Evaluation Scale III (FACES III: Olson et al ., 1985);<br>2) Positive and Negative Suicide Ideation Inventory (PANSI: Osman et al ., 1998)		1) dimensions of family functioning - family cohesion ($r = -0.171$, $p < 0.001$) and family flexibiliy ($r = -0.083$, $p < 0.05$) were significantly and negatively associated with SI;<br>2) based on mediation analysis with problem-focused coping as a mediator, it did not mediate the association between family cohesion and SI (b = −0.011, SE = 0 .006, 95% CI [−0.022, 0.001]), however, it mediated the association between family flexibility and SI (b = −0.014, SE = 0 .004, 95% CI [−0.022, −0.006]);<br>3) the significant mediation model indicates that family flexibility has no significant direct effect on SI. This means that adolescents with families who have a weaker ability to adapt to family changes might be less likely to engage in problem-focused coping strategies, which then contributes to more SI.
Lee et al., (2016), Vietnam	N = 169	1) 82 males (48.5%);<br>2) 87 females (51.5%)	12 years old (95.9%) (M = , SD =)	Cross-sectional survey	1) parental drinking,<br>2) expecting too much by parents	1) checking homework by parents,<br>2) understanding problems and worries with parents' help,<br>3) having free time from parents,<br>4) doing something without parents' approval,<br>5) giving advice and guidance by parents,<br>6) parents respecting the student as a person	1) GSHS questionnaire items developed by the WHO (WHO, 2013)	1) among all the family-related variables, only adolescents with parents who drink alcohol had a higher risk of SI (OR=3.72; 95% CI [1.04, 13.35])	
Kwok & Shek, (2008), Hong Kong	N = 5557	1) males ();<br>2) females ()	11-18 years (M = , SD =)	Cross-sectional survey	1) Family economic status;<br>2) Family work status;<br>3) Family structure;<br>4) Family educational status		1) Suicidal Ideation Sub-Scale (CSIS) of the Suicidal Risk Scale for Hong Kong students developed locally by Tse, 2002;<br>2) the socio-demographic correlates of adolescent suicidal ideation, the participants were requested to provide information based on the studied variables.	1) adolescents living with intact family reported low SI as compared to those not living with intact family ($F(l, 5244) = 51.53$, $p < 0.001$, partial η^2 =. 010);<br>2) adolescents reported high SI with family who received financial aid from the government called Comprehensive Social Security Allowance (CSSA) ($F(l, 5167) = 17.10$, $\rho <.001$ .partial $\eta^2 = .003$);<br>3) both mother ($F(3, 5127) = 5.64$, $p < .01$, partial $\eta^2 = .001$) and father ($F(3, 4990) = 4.44$, $p<.01$, partial $\eta^2 = .002$) with different work status showed strong association with different levels of SI;<br>4) adolescents who had fathers ($F (2, 5027)= 15.73$, $p < 0.001$, partial $\eta^2 = .006$) with different educational levels had significant differences in SI, whereas higher education level of mothers was associated with low SI ($F (2,5072) = 6.24$, $p < 0.01$, partial $\eta^2 = .002$)	
Herba et al., (2008), Netherlands	N = 1526	1) males ();<br>2) females ()	Time 1 - (M = 11.09, SD = 0.55);<br>Time 2 - (M = 13.56, SD = 0.53)	Cohort study	1) Rejection at home;<br>2) Parental psychopathology		1) EMBU-C (Swedish acronym for 'My Memories of Upbringing'; Markus, Lindhout, Boer, Hoogendijk, & Arrindell, 2003) - rejection at home;<br>2) TRAILS Family History Interview at T1 - Parental psychopathology;<br>3) Youth Self Report (YSR) (Achenbach, 1991) - SI	1) based on logistic regression, rejection at home (OR = 6.42, 95% CI [4.72, 8.73], $p < 0.001$), and parental internalizing disorder (OR = 1.18, 95% CI [1.03, 1.36], $p <0.02$) had a significant association with SI;<br>2) with parental rejection and internalizing disorders used as moderator, the interation of victimization and parental rejection (Wald = 5.90, df = 2, p=.05) and interaction between victimization and internalizing disorders (Wald = 5.90, df = 2, p=.05) significantly moderated the impact on SI.	
Park et al., (2005), South Korea	N = 501	1) 271 males (54.1%);<br>2) 230 females (45.9%)	(M = , SD =)	Cross-sectional survey		Family communication	1) Korean version of the PACI (Parent-Adolescent Communication Inventory), which was developed by Barnes and Olson (1985) and translated by Min (1990) - Family communication;<br>2) Korean version of the SSI-C (Scale for Suicide Ideation), by Beck et al., 1979, and translated by Park and Sin (1991)	1) family communication associated both significantly and negatively with SI for male ($r = -0.366$, $p < 0.001$) and female adolescents ($r = -0.302$, $p < 0.001$);<br>2) based on multiple regression analysis, family communication was a significant predictor of SI among males only in both models ($\beta = -0.15$, $t = 2.17$, $p = .031$)	

Ang & Ooi, (2004), Singapore	N = 271	1) 149 males (54.98%); 2) 122 females (45.02%)	(M = 14.12, SD = 0.94)	Cross-sectional survey	Parental marital status		1) Participants were asked to provide their parents' marital status based on five options - Married, Separated, Divorced, Never Married/Single, and Widowed; 2) Suicidal Ideation Questionnaire-junior high school version (SIQ-JR) by Reynolds, 1988.	1) Based on ANOVA, the main effect of parental marital status on SI was not significant (F (1265) = 3.10, p = 0.08); 2) simple effect tests were performed based on Tukey's test, and boys from single-parent families reported higher SI (n = 18, M = 29.50, SD = 22.30) as compared to boy from two parent families (n = 129, M = 13.39, SD = 21.20).	
De Man et al., (2010), Canada	N = 558	1) 272 males (48.7%); 2) 286 females (51.3%)	11-18 years (M = 14, SD = 1.46)	Cross-sectional survey	1) Parental marital status; 2) Parental control	1) parental support	1) parental marital status was asked directly using questionnaires; 2) Autonomy-Control Scale (de Man, 1987-1988; de Man et al., 1991) - Parental control; 3) Social Support Questionnaire (de Man, Balkou, & Iglesias, 1986; Sarason. Levine. Basham, & Sarason, 1983); 4) Scale for Suicide Ideation (Beck, Kovacs. & Weissman, 1979) - French version	1) parental marital status did not have a significant association with SI; 2) high parental control had a significant negative association with SI (r = -0.14, p < 0.001);	1) adolescents with SI did not consider their mother (r = -0.32, p < 0.001) or father (r = -0.30, p < 0.001) as a source of support; 2) based on multivariate analysis, maternal support was the only significant predictor of SI (F(1, 556) = 63.2, p < .001), accounting for a variance of 10.2%
Low, (2021), Hong Kong	N = 338	1) 143 males (42.3%); 2) 195 females (57.7%)	(M = 13.41, SD = 1.13)	Cross-sectional survey	Parent-adolescent conflict		1) Conflict Behaviour Questionnaire (CBQ-20) by Robin and Foster (1989) - Parent-adolescent conflict; 2) Suicidal Risk Scale for Hong Kong Students (Tse et al., 2002) - SI	1) Parent-adolescent conflict had a significant and positive association with SI (r = 0.474, p < 0.01); 2) based on regression analysis, mediating effect of depression and anxiety between parent-adolescent conflict and SI was measured, both depression (B =0.389, SE = 0.150, 95% CI [0.214, 0.576]) and anxiety (B = 0.386, SE = 0.092, 95% CI [0.214, 0.576]) mediated the association.	
Lai & McBride-Chang (2001), Hong Kong	N = 120	1) 45 males (37.5%); 2) 75 females (62.5%)	15-19 years (M = 16.1, SD = 1.6)	Cross-sectional survey		1) Parenting style; 2) Family climate	1) Parental Authority Questionnaire (PAQ; Buri, 1991) - Parenting styles; 2) Parental Bonding Instrument (PBI; Parker, Tupling, & Brown, 1979); 3) Parenting practice questionnaire (Chen, 1994); 4) Family Climate Questionnaire (Kurdek & Fine, 1993); 5) Scale of Suicide Ideation (SSI; A. Beck et al., 1979)		1) the mean scores for mother authoritarian (M = 30.11, SD = 5.53), father authoritarian (M = 30.11; SD = 5.89), and mother over-control (M =15.79, SD = 5.65) parenting styles were higher for those with SI, when compared with those who did not have SI (M = 27.40, SD = 5.60; M = 26.31, SD = 5.00; M = 12.57, SD = 4.65; respectively); 2) based on t-tests, those with SI perceived more conflict (M = 15.92; SD = 6.77, t = 3.13) and less warmth (M = 22.79; SD = 5.63, t = 3.72) in the family as compared to non-ideators ((M = 12.24; SD = 6.04, conflict; M = 6.17; SD = 4.17, warmth)
Shagle & Barber, (1993), USA	N = 473	1) 213 males (45%); 2) 260 females (55%)	(M = 13.7, SD =)	Cross-sectional survey	1) Family conflict; 2) Parent-adolescent conflict; 3) Marital conflict		1) Colorado Self-Report of Family Functioning Inventory (Bloom, 1985) - Family conflict; 2) National Survey of Families and Households (Sweet, Bumpass, & Call, 1988) - Parent-adolescent conflict; 3) marital conflict scale by Emery and O'Leary's (1982) - marital conflict; 4) Beck Depression Scale (Beck, 1978) - SI	1) Family conflict (r = 0.21, p < 0.01), parent-adolescent conflict (r = 0.19, p < 0.01), and marital status (r = 0.26, p < 0.01) had a significant positive association with SI; 2) based on linear structural analysis, it was reported that self-derogation as a mediator significantly mediated the association between family conflict (2.6, p < 0.01) and SI, and parent-adolescent conflict (2.1, p < 0.05) and SI, but not between marital conflict and SI (1.4, p > 0.05)	
Hockenberry et al., (2010), USA	N = 575	1) 287 males (49.9%); 2) 288 females (50.1%)	14-19 years	Cross-sectional survey	Parents smoking		1) National Annenberg Survey of Youth (NASY)	1) The multivariate regression analysis reported that participants with one or both parents who smoked did not significantly predict SI (OR = 1.26; 95% CI = 0.37–4.23).	
