# Supplementary material for: The impact of family-related psychosocial factors on suicidal ideation among adolescents: a systematic review
Source: BMC Public Health. 2026 Jul 9;26:2743. doi: 10.1186/s12889-026-27928-7 (PMC13632444; doi:10.1186/s12889-026-27928-7)
Supplement: Supplementary file 2 [file 12889_2026_27928_MOESM2_ESM.pdf]

Cross-sectional & Longitudinal Studies													
**Study Number**	**Study (Author, Year)**	**Were the criteria for inclusion in the sample clearly defined?**	**Were the study subjects and the setting described in detail?**	**Was the exposure measured in a valid and reliable way?**	**Were objective, standard criteria used for measurement of the condition?**	**Were confounding factors identified?**	**Were strategies to deal with confounding factors stated?**	**Were the outcomes measured in a valid and reliable way?**	**Was appropriate statistical analysis used?**	**Overall Appraisal**			
54	Picou et al., (2024), USA	Yes	Yes	Yes	Yes	No	No	Yes	Yes	Include			
64	Keshky, (2024), Saudi Arabia	Yes	Yes	Yes	Yes	No	No	Yes	Yes	Include			
88	Buelga, (2024), Spain	Yes	Yes	Yes	Yes	Yes	Yes	Yes	Yes	Include			
101	Ding et al., (2022), China	Yes	Yes	Yes	Yes	Yes	Yes	No	Yes	Include			
174	Sun et al., (2022), China	No	Yes	Yes	Yes	No	No	Yes	Yes	Include			
298	Lee et al., (2021), South Korea	Yes	Yes	Yes	Yes	Yes	Yes	No	Yes	Include			
361	Handley et al., (2019), USA	Yes	Yes	Yes	Yes	No	No	Yes	Yes	Include			
539	Lardier et al. (2016), USA	Yes	Yes	Yes	Yes	No	No	Yes	Yes	Include			
640	Kwok et al., (2015), China	Yes	Yes	Yes	Yes	Yes	Yes	Yes	Yes	Include			
784	Kwok et al., (2013), China	Yes	Yes	Yes	Yes	Yes	Yes	Yes	Yes	Include			
807	Samm et al., (2009), Estonia	Yes	Yes	Yes	Yes	No	No	Yes	Yes	Include			
827	Kwok & Shek, (2011), China	Yes	Yes	Yes	Yes	Yes	Yes	Yes	Yes	Include			
837	Mahfoud et al., (2010), Lebanon	Yes	Yes	Yes	Yes	Yes	Yes	Yes	Yes	Include			
848	An et al., (2010), South Korea	Yes	Yes	Yes	Yes	Yes	Yes	Yes	Yes	Include			
849	Logan et al., (2011), USA	Yes	Yes	Yes	Yes	Yes	Yes	No	Yes	Include			
1054	Springer et al., (2005), USA	Yes	Yes	Yes	Yes	Yes	Yes	Yes	Yes	Include			
1067	Lee et al., (2006), China	Yes	Yes	Yes	Yes	No	No	Yes	Yes	Include			
1173	Shilubane et al., (2013), South Africa	Yes	Yes	Yes	Yes	No	No	Yes	Yes	Include			
1176	Yang et al., (2022), China	Yes	Yes	Yes	Yes	Yes	No	Yes	Yes	Include			
1179	Yoder & Hoyt, (2005), USA	Yes	Yes	Yes	Yes	Yes	No	No	Yes	Include			
1189	Yang et al., (2023), China	Yes	Yes	Yes	Yes	Yes	Yes	Yes	Yes	Include			
1194	Peltzer & Pengpid, (2012), Thailand	Yes	Yes	Yes	Yes	No	No	No	Yes	Include			
1199	Li et al., (2016), China	Yes	Yes	Yes	Yes	No	No	Yes	Yes	Include			
1209	Lee & Choi, (2015), South Korea	Yes	Yes	Yes	Yes	No	No	Yes	Yes	Include			
1212	Estévez-García et al., (2023), Spain	Yes	Yes	Yes	Yes	Yes	Yes	Yes	Yes	Include			
1224	Lee et al., (2018), South Korea	Yes	Yes	Yes	Yes	No	No	Yes	Yes	Include			
1226	Wan et al., (2022), China	Yes	Yes	Yes	Yes	Yes	No	Yes	Yes	Include			
1227	Chae et al., (2020), South Korea	Yes	Yes	Yes	Yes	Yes	No	Yes	Yes	Include			
1231	Peltzer & Pengpid, (2012), South Africa	Yes	Yes	Yes	Yes	No	No	Yes	Yes	Include			
1267	Peng et al., (2024), China	Yes	Yes	Yes	Yes	No	No	Yes	Yes	Include			

1289	Kwok et al., (2009), China	Yes	Yes	Yes	Yes	No	No	Yes	Yes	Include			
1373	Asante et al., (2021), Sierra Leone	Yes	Yes	Yes	Yes	No	No	Yes	Yes	Include			
1397	Bao et al., (2024), China	Yes	Yes	Yes	Yes	No	No	Yes	Yes	Include			
1435	Yang et al., (2023), China	Yes	Yes	Yes	Yes	No	No	Yes	Yes	Include			
1449	Walsh et al., (2012), Israel	Yes	Yes	Yes	Yes	No	No	Yes	Yes	Include			
1490	Pin˜a-Watson et al., (2014), USA	Yes	Yes	Yes	Yes	No	No	No	Yes	Include			
1503	Gouveia-Pereira et al., (2014), Portugal	Yes	Yes	Yes	Yes	No	No	Yes	Yes	Include			
1566	Guan et al., (2024), China	Yes	Yes	Yes	Yes	Yes	No	Yes	Yes	Include			
1569	Cho, (2020), South Korea	Yes	Yes	Yes	Yes	No	No	Yes	Yes	Include			
1575	Li et al., (2025), China	Yes	Yes	Yes	Yes	Yes	Yes	Yes	Yes	Include			
1578	Potard et al., (2020), France	Yes	Yes	Yes	Yes	No	No	Yes	Yes	Include			
1593	Guo et al., (2021), China	Yes	Yes	Yes	Yes	No	No	Yes	Yes	Include			
1636	Wang & Qiao, (2022), China	No	Yes	Yes	Yes	No	No	Yes	Yes	Include			
1638	Wang et al., (2014), Taiwan	Yes	Yes	Yes	Yes	Yes	Yes	Yes	Yes	Include			
1668	Bar-Zomer & Brunstein Klomek, (2018), Israel	Yes	Yes	Yes	Yes	Yes	No	Yes	Yes	Include			
1679	Chen et al., (2020), China	Yes	Yes	Yes	Yes	Yes	Yes	Yes	Yes	Include			
1687	Wang et al., (2023), China	Yes	Yes	Yes	Yes	No	No	Yes	Yes	Include			
1698	Kim et al., (2021), South Korea	Yes	Yes	Yes	Yes	No	No	Yes	Yes	Include			
1707	Kwok & Shek, (2009), China	Yes	Yes	Yes	Yes	No	No	Yes	Yes	Include			
1728	Wu & Yaccob, (2016), Malaysia	Yes	Yes	Yes	Yes	Yes	Yes	Yes	Yes	Include			
1738	Nunes & Mota, (2016), Portugal	Yes	Yes	Yes	Yes	No	No	Yes	Yes	Include			
1746	Kelly et al., (2025), USA	Yes	Yes	Yes	Yes	Yes	Yes	Yes	Yes	Include			
1758	Nieto-Casado et al., (2022), Spain	Yes	Yes	Yes	Yes	Yes	Yes	Yes	Yes	Include			
1766	Nakano, (2021), Japan	Yes	Yes	Yes	Yes	No	No	Yes	Yes	Include			
1770	Modeste-James et al., (2023), USA	Yes	Yes	Yes	Yes	Yes	Yes	Yes	Yes	Include			
1818	Peláez-Fernández et al., (2024), Spain	Yes	Yes	Yes	Yes	Yes	Yes	Yes	Yes	Include			
1834	Ibrahim et al., (2019), Malaysia	Yes	Yes	Yes	Yes	Yes	Yes	Yes	Yes	Include			
1849	Kim et al., (2021), USA	Yes	Yes	Yes	Yes	Yes	Yes	Yes	Yes	Include			
1874	Wang et al., (2023), China	Yes	Yes	Yes	Yes	Yes	Yes	Yes	Yes	Include			
1877	Galindo-Dominguez & Losada Iglesias, (2023), Spain	Yes	Yes	Yes	Yes	No	No	Yes	Yes	Include			
1883	Eslava et al., (2023), Spain	Yes	Yes	Yes	Yes	Yes	Yes	Yes	Yes	Include			
1929	Quarshie et al., (2023), Namibia	Yes	Yes	Yes	Yes	Yes	Yes	Yes	Yes	Include			
1945	Bao et al., (2020), China	Yes	Yes	Yes	Yes	No	No	No	Yes	Include			

1969	Arango et al., (2018), USA	Yes	Yes	Yes	Yes	Yes	Yes	Yes	Yes	Include			
2051	Ahouanse et al., (2022), China	Yes	Yes	Yes	Yes	Yes	Yes	Yes	Yes	Include			
2165	Stone et al., (2015), USA	No	Yes	Yes	Yes	Yes	Yes	No	Yes	Include			
2227	Sarfo et al., (2023), Saint Lucia	No	Yes	Yes	Yes	No	No	Yes	Yes	Include			
2344	Rubio et al., (2020), Chile	No	Yes	Yes	Yes	No	No	Yes	Yes	Include			
2849	Kaminski et al., (2009), USA	No	Yes	Yes	Yes	Yes	Yes	Yes	Yes	Include			
3055	Barzilay et al., (2017), European countries (Austria, Estonia, France, Germany, Hungary, Ireland, Italy, Romania, Slovenia, and Spain)	No	Yes	Yes	Yes	Yes	Yes	Yes	Yes	Include			
3237	Ai et al., (2017), China	No	Yes	Yes	Yes	Yes	Yes	Yes	Yes	Include			
3344	Caballero-Domınguez & Campo-Arias, (2022), Colombia	Yes	Yes	Yes	Yes	Yes	Yes	Yes	Yes	Include			
3589	Goodwill, (2020), USA	No	Yes	Yes	Yes	Yes	Yes	Yes	Yes	Include			
3633	Jung & Cho, (2020), South Korea	Yes	Yes	Yes	Yes	Yes	Yes	Yes	Yes	Include			
3759	Liu et al., (2024), China	No	Yes	Yes	Yes	Yes	Yes	Yes	Yes	Include			
3827	King et al., (2018), USA	No	Yes	Yes	Yes	No	No	Yes	Yes	Include			
4001	Sun & Ban, (2022), China	No	Yes	Yes	Yes	No	No	Yes	Yes	Include			
5252	Mulenga et al., (2017), Morocco	No	Yes	Yes	Yes	No	No	Yes	Yes	Include			
5257	Siziya et al., (2017), Samoa	No	Yes	Yes	Yes	No	No	Yes	Yes	Include			
5546	Aliy et al., (2023), Ethiopia	Yes	Yes	Yes	Yes	No	No	Yes	Yes	Include			
5596	Han et al., (2023), South Korea	Yes	Yes	Yes	Yes	Yes	Yes	Yes	Yes	Include			
5627	Wu et al., (2023), China	Yes	Yes	Yes	Yes	Yes	Yes	Yes	Yes	Include			
6225	Cong et al., (2020), Malaysia	No	Yes	Yes	Yes	No	No	Yes	Yes	Include			
6777	Lee et al., (2016), Vietnam	No	Yes	Yes	Yes	No	No	Yes	Yes	Include			
7665	Kwok & Shek, (2008), Hong Kong	Yes	Yes	Yes	Yes	No	No	Yes	Yes	Include			
7865	Park et al., (2005), South Korea	Yes	Yes	Yes	Yes	No	No	Yes	Yes	Include			
7918	Ang & Ooi, (2004), Singapore	No	Yes	Yes	Yes	No	No	Yes	Yes	Include			
8271	De Man et al., (2010), Canada	No	Yes	Yes	Yes	No	No	Yes	Yes	Include			
8797	Low, (2021), Hong Kong	No	Yes	Yes	Yes	Yes	Yes	Yes	Yes	Include			
9878	Lai & McBride-Chang (2001), Hong Kong	No	Yes	Yes	Yes	No	No	Yes	Yes	Include			
10023	Shagle & Barber, (1993), USA	No	Yes	Yes	Yes	No	No	Yes	Yes	Include			
**Randomized Controlled Trial (Study no. 1688, MacPherson et al., (2021), USA)**													
**Item**	**Assessment**	**Justification**	**Overall Appraisal**										

Was true randomization used for assignment of participants to treatment groups?	Yes	Two-group randomized parallel trial, families were assigned to I-CBT or TAU after baseline.	Include										
Was allocation to treatment groups concealed?	Unclear	The report does not describe concealment.											
Were treatment groups similar at the baseline?	Yes	Baseline characteristics described, no dissimilarity noted.											
Were participants blind to treatment assignment?	No	TAU makes blinding impractical											
Were those delivering the treatment blind to treatment assignment?	No	The researchers were aware of the treatment provided.											
Were treatment groups treated identically other than the intervention of interest?	Unclear	Information providing such details missing.											
Were outcome assessors blind to treatment assignment?	Yes	Follow-up assessors were blind to allocation.											
Were outcomes measured in the same way for treatment groups?	Yes	Same tools were used.											
Were outcomes measured in a reliable way	Yes	Both the measures showed high internal consistency in the sample.											
Was follow up complete and if not, were differences between groups in terms of their follow up adequately described and analysed?	Yes	Attrition was recorded, and analysis was done with missing data in consideration.											
Were participants analysed in the groups to which they were randomized?	Yes	Mixed-effects models kept participants in assigned arms under MAR.											
Was appropriate statistical analysis used?	Yes	Longitudinal mixed-effects with covariance and covariates.											

Was the trial design appropriate and any deviations from the standard RCT design (individual randomization, parallel groups) accounted for in the conduct and analysis of the trial?	Yes	Standard individually randomized parallel design.											
**Cohort Studies**													
**Study Number**	**Study (Author, Year)**	**Were the two groups similar and recruited from the same population?**	**Were the exposures measured similarly to assign people to both exposed and unexposed groups?**	**Was the exposure measured in a valid and reliable way?**	**Were confounding factors identified?**	**Were strategies to deal with confounding factors stated?**	**Were the groups/participants free of the outcome at the start of the study (or at the moment of exposure)?**	**Were the outcomes measured in a valid and reliable way?**	**Was the follow up time reported and sufficient to be long enough for outcomes to occur?**	**Was follow up complete, and if not, were the reasons to loss to follow up described and explored?**	**Were strategies to address incomplete follow up utilized?**	**Was appropriate statistical analysis used?**	**Overall appraisal**
7702	Herba et al., (2008), Netherlands	Yes	Yes	Yes	Yes	Yes	Unclear	Yes	Yes	Yes	Yes	Yes	Include
