# Supplementary material for: The impact of family-related psychosocial factors on suicidal ideation among adolescents: a systematic review
Source: BMC Public Health. 2026 Jul 9;26:2743. doi: 10.1186/s12889-026-27928-7 (PMC13632444; doi:10.1186/s12889-026-27928-7)
Supplement: Supplementary file 3 [file 12889_2026_27928_MOESM3_ESM.doc]

**Table 1 Year (number of studies), included studies**

| **Year (n)** | **Included Studies** |
| --- | --- |
| 2025 (2) | (36,37) |
| 2024 (8) | (38–45) |
| 2023 (13) | (46–58) |
| 2022 (9) | (59–67) |
| 2021 (8) | (68–75) |
| 2020 (9) | (76–84) |
| 2019 (2) | (85,86) |
| 2018 (4) | (87–90) |
| 2017 (4) | (91–94) |
| 2016 (5) | (95–99) |
| 2015 (3) | (100–102) |
| 2014 (3) | (103–105) |
| 2013 (2) | (106,107) |
| 2012 (3) | (108–110) |
| 2011 (2) | (111,112) |
| 2010 (4) | (113–116) |
| 2009 (4) | (117–120) |
| 2008 (2) | (121,122) |
| 2007 (0) |  |
| 2006 (1) | (123) |
| 2005 (3) | (124–126) |
| 2004 (1) | (127) |
| 2003 (0) |  |
| 2002 (0) |  |
| 2001 (1) | (128) |
| 2000 (0) |  |
| 1993 (1) | (129) |

Table 2 Region, country, and included studies.

| **Region** | **Country** | **Included Studies** |
| --- | --- | --- |
| North America (19) | USA (18) | (37,38,50,71,74,82,85,89,90,95,102,103,112,116,120,124,125,129) |
|  | Canada (1) | (115) |
|  | Saint Lucia (1) | (55) |
| South America (2) | Chile (1) | (81) |
|  | Colombia (1) | (66) |
| Europe (12) | 10 European countries (1) | (91) |
|  | France (1) | (78) |
|  | Spain (6) | (40,44,47,51,53,64) |
|  | Netherlands (1) | (122) |
|  | Estonia (1) | (117) |
|  | Portugal (2) | (98,104) |
| Asia (53) | South Korea (10) | (57,68,74,76,77,83,87,101,114,126) |
|  | China (28) | (36,41–43,45,46,48,49,52,58–63,65,67,70,79,80,92,96,100,106,111,118,119,123) |
|  | Hong Kong (3) | (75,121,128) |
|  | Taiwan (1) | (105) |
|  | Vietnam (1) | (99) |
|  | Malaysia (3) | (84,86,97) |
|  | Singapore (1) | (127) |
|  | Israel (2) | (88,110) |
|  | Thailand (1) | (108) |
|  | Saudi Arabia (1) | (39) |
|  | Japan (1) | (73) |
|  | Lebanon (1) | (113) |
| Africa (6) | South Africa (2) | (107,109) |
|  | Ethiopia (1) | (56) |
|  | Morocco (1) | (93) |
|  | Sierra Leone (1) | (69) |
|  | Namibia (1) | (54) |
| Oceania (1) | Samoa (1) | (94) |

Table 3 Outcome

| **Sl No.** | **Domains** | **Citations** |
| --- | --- | --- |
| 1 | Hostility inside the family | (38,49,51,66,68,72,75,77,82,85,87,88,92,95,100,104,106,107,122,123,125,129) |
| 2 | Family Communication | (40,44,46,59,61,64,70,71,78,80,85,88,89,105,106,108,111,114,117–120,128) |
| 3 | Attachment with family members | (39,46,58,59,70,78,80,85,88,89,97,98,102,108,114,117,120,123,123,130) |
| 4 | Parental emotional availability | (37–39,41,42,48,50,52–56,59,62,63,65,68,69,73,77,81–83,86,90,91,93,94,96,99,103,107,109,110,112,113,115,123,124) |
| 5 | Parental control | (36,39,43,47,50,54,60,67,69,71,74,77,98,103,112,115,128) |
| 6 | Parental psychopathology | (56,57,62,76,122,125) |
| 7 | Parental socioeconomic and educational status | (45,46,79,96,101,115,117,120,121,127,129) |
| 8 | Parental substance use | (55,99,116) |

Table 4 Outcomes of the studies included

| **Category** | **Outcome** | **Studies** |
| --- | --- | --- |
| Hostility inside the family | Parent-child conflict (1), Family conflict (4), Parent-adolescent conflict (3), Parental conflict (1), Mother-adolescent conflict (1), Interparental conflict (1), Conflictual independence (1) | (38,51,75,82,85,87,92,95,104,123,129) |
|  | Family dysfunction (2) | (66,68) |
|  | Parental abuse (2), Physical abuse (2), Psychological abuse (1), Parental hostile behavior (1), Family violence (1), Parental neglect (1), Sibling bullying (1), Rejection at home (1), Family incivility (1) | (49,68,77,88,100,106,122,125) |
|  | Negative feelings towards family (1) | (107) |
| Family Communication | Family communication (3), Parent-adolescent communication (2) | (40,64,111,119,126) |
|  | Family functioning (8) | (61,71,84,104–106,111,118) |
| Attachment with family members | Satisfaction with family (1), subjective satisfaction with relationships among family members (1), perceived parents' dissatisfaction (1) | (114,117,123) |
|  | Family cohesion (3), Parental closeness (1), Family connectedness (4), Family togetherness (1) | (39,58,80,89,97,102,103,120,123) |
|  | Parent-child attachment relationship (1), Parental attachment (5), Perceived relationship quality with parents (1), Mother-Adolescent Relationship Quality (1) | (46,59,70,78,85,88,98,108) |
| Parental emotional availability | Perceived social support from family (4), Perceived family support (1), Family social support (3), Family support (4), Support from family (1), Parental social support (1), Perceived parental social support (1), Parental support (6), Lack of parental support (1), Supportive parents’ responses to children’s negative emotions (PRCNE) (1) | (37,39,41,42,48,53,59,62,65,68,73,81,83,86,91,93,96,107,109,110,115,123,124) |
|  | Familism (1) | (38) |
|  | Parental understanding (7), Understanding parents (1) | (50,54–56,69,93,94,113) |
|  | Verbal affirmation from parents (1), Perceived acceptance from mother (1), expecting too much by parents (1) | (52,82,99) |
|  | Parental involvement (1), Parental academic interest (1), Checking homework by parents (1), Parents’ participation in education (1), Parental phubbing (1), Mother phubbing (1) | (52,63,77,90,99,103) |
|  | Parental care (2) | (103,112) |
| Parental control | Parental psychological control (2), Parental control (1) | (36,67,115) |
|  | Parental monitoring (4), Parental supervision (4), Autonomy (1) | (54,69,71,74,77,103,112) |
|  | Parental invasion of privacy (1), Parental intrusion of privacy (2) | (50,54,69) |
|  | Positive parenting (1), Negative parenting style (2), Harsh parenting (1), Parenting Style (2) | (39,43,47,60,98,128) |
| Parental psychopathology | Parental psychopathology (1), Parental depressive mood (1), Parental depressive symptoms (1), | (57,122,125) |
|  | Parental suicidal ideation(2), Family suicide history (2), Family history of suicide attempts (1) | (56,57,62,76) |
| Parental socioeconomic and educational status | Parental marital status (3), Marital conflict (1), Family structure (3), Socio-economic status (2), Family economic deprivation (1), Family economic status (1), Family economic pressure (1), Family work status (1), Parents’ work stress (1), Family financial stress (1) | (45,46,79,96,101,115,117,120,121,127,129) |
|  | Parent education (1), Family educational status (1) | (101,121) |
| Parental substance use | Parental use of tobacco (1), Parental drinking (1), Parent/s smoking | (55,99,116) |
